# Engineering Chiroptical Interactions through Integrating Plasmonic Arrays with Cholesteric Nanocellulose

**DOI:** 10.1002/adma.202519964

**Published:** 2026-02-15

**Authors:** Han Tao, Sunghwan Jo, Guang Chu, Xiaoyu Qi, Irene Estévez, Angel Lizana, Wenyang Xu, Shengwei Deng, Agustin Mihi, Eero Kontturi

**Affiliations:** ^1^ Department of Bioproducts and Biosystems Aalto University School of Chemical Engineering Espoo Finland; ^2^ Institute of Materials Science of Barcelona ICMAB‐CSIC Bellaterra Spain; ^3^ School of Chemistry and Chemical Engineering Southeast University Nanjing China; ^4^ Grup d’Òptica Departament de Física Universitat Autònoma de Barcelona UAB Bellaterra Spain; ^5^ Max Planck Institute of Colloids and Interfaces Potsdam Germany; ^6^ College of Chemical Engineering Zhejiang University of Technology Hangzhou China

**Keywords:** cellulose nanocrystals, chiral light‐matter interaction, chiral plasmonics, plasmonic metasurface

## Abstract

Achieving scalable fabrication with precise control of chiroptical properties in chiral plasmonic materials remains challenging. We present a new family of engineered chiroptical composites comprising linearly assembled gold nanoparticle arrays integrated with cholesteric self‐assembled cellulose nanocrystals (CNCs). Aqueous CNC suspensions are cast onto pre‐assembled achiral plasmonic nanoparticle arrays via evaporation‐induced transfer imprinting lithography, yielding centimeter‐scale hybrid films with custom‐tailored chiroptical responses. During drying, CNCs co‐assemble with the gold nanoparticles at the interface, preserving the array's linear arrangement and keeping it isolated from the overlying cholesteric CNC layers. This configuration combines the linear dichroism of the plasmonic array with the linear birefringence of the CNC matrix, producing strong and tunable plasmonic circular dichroism at the surface lattice resonance, reaching 1217 ± 51 mdeg with a dissymmetry factor of –0.19 ± 0.02. Our approach provides a sustainable platform for engineering multifunctional chiral plasmonic materials with potential applications in optical sensing, photonic devices, and chiral biointerfaces.

## Graphical abstract

In this study, we demonstrate how the combination of plant‐based cellulose and gold nanoparticle lines produces robust, tunable chiral light control in composite films. The resulting plasmonic cellulose composite exhibits a strong plasmonic CD at the surface lattice resonance, along with an inversion of chiroptical response upon reversing the illumination direction, revealing an antisymmetric CD behavior.

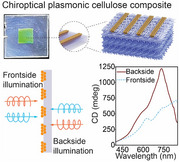

## 1 Introduction

Chirality describes the geometric property of an object that cannot be superimposed on its mirror image by rotation or translation—a fundamental aspect in both natural and synthetic systems [1, 2]. It is ubiquitous across multiple length scales and various scientific disciplines, from chemistry and physics to biology and materials science, playing a key role in controlling molecular behavior, biological activity, and material functionality [3, 4, 5, 6]. Chiroptical phenomena, on the other hand, emerge when chiral materials interact with light, specifically with left‐ and right‐handed circularly polarized light (LCP and RCP) [7, 8, 9]. Engineering chiroptical interactions bears significant potential for applications in advanced sensors, [10, 11] catalysts, [12] cryptographic optical devices, [13, 14] and quantum photonics. [15] Unfortunately, conventional chiral materials show weak and narrow chiroptical responses, and, in consequence, intricate ways to enhance the response have surfaced, particularly by coupling chiral structures with plasmonic nanoparticles. [16, 17, 18, 19, 20, 21] This study lays out a unique approach to chiroptical interactions by utilizing the birefringence of chiral cellulose nanocrystals (CNCs) as a basis for a plasmonic metasurface made of gold nanoparticles (AuNPs). It marks the advent of applying the intrinsic birefringence of natural, plant‐based structures in the design of chiroptical materials.

A plasmonic metasurface is a specifically engineered 2D planar array of metal nanostructure that can manipulate light through surface plasmon resonance [22, 23, 24]. For example, LCP light illumination strongly excites the surface plasmons, leading to emphasized absorption or reflection, whereas RCP light interaction is much weaker [25, 26, 27]. Such a difference in interaction gives rise to pronounced chiroptical responses that can be finely tuned through the design of geometry, periodicity, and material composition [28, 29, 30, 31, 32]. Specifically, combining plasmonic metasurfaces that exhibit linear dichroism (LD) with linearly birefringent (LB) substrates can result in strong extrinsic chirality at specific wavelengths [33, 34]. At present, all efforts toward LD‐LB sequential optical configuration have involved the use of synthetic polymer substrates where constant strain is applied to maintain the desired LB activity. Such systems face several limitations, including poor long‐term mechanical stability and degraded optical performance, as the constant strain can result in material fatigue. By contrast, CNCs—top‐down isolated from plant fibers—are intrinsically chiral nanorods which possess strong optical anisotropy by default [35, 36, 37, 38, 39, 40, 41, 42]. They self‐assemble into liquid crystal domains with a helicoidal structure that can be retained in macroscopic dried films, where the birefringence of individual CNCs (Δ*n*_CNC_ = 0.072) is preserved, albeit at a slightly reduced value (Δ*n*_film_ = 0.062) due to imperfect CNC alignment [43, 44]. This unique combination of birefringence and chiral nematic organization results in tunable photonic bandgap (PBG), which has been utilized in vivid structural coloration of the obtained films, among many other examples of photonic devices [45, 46, 47, 48, 49, 50, 51, 52].

This study demonstrates a new type of plasmonic metasurface compositing linearly assembled AuNP arrays supported by cholesteric CNC films, showing LB‐LD chiroptical response. These hybrid composites are fabricated by casting aqueous CNC suspensions onto pre‐assembled AuNP arrays via an evaporation‐assisted transfer imprinting approach. During the drying process, the rodlike CNC nanoparticles self‐assemble into cholesteric organization over the plasmonic arrays, which remain localized at the solid‐liquid interface. This results in centimeter‐scale films with a multiscale photonic architecture, where planar plasmonic arrays reside at the film surface, and cholesteric order prevails throughout the bulk matrix. The obtained plasmonic nanocellulose composite films exhibit strong and spectrally tunable CD at the surface lattice resonance (SLR), with a maximum value of 1217 ± 51 mdeg and a dissymmetry factor of −0.19 ± 0.02. Mueller matrix (MM) polarimetry further reveals that the pronounced chiroptical activity originates from the synergistic interplay between the LD‐active plasmonic grating and phase retardance provided by the birefringent CNC matrix. Our approach delivers a sustainable, scalable route to create hierarchical photonic materials, paving the way for multifunctional optical platforms that unite sustainability, biocompatibility, and advanced photonic performance.

## 2 Results

**FIGURE 1:**
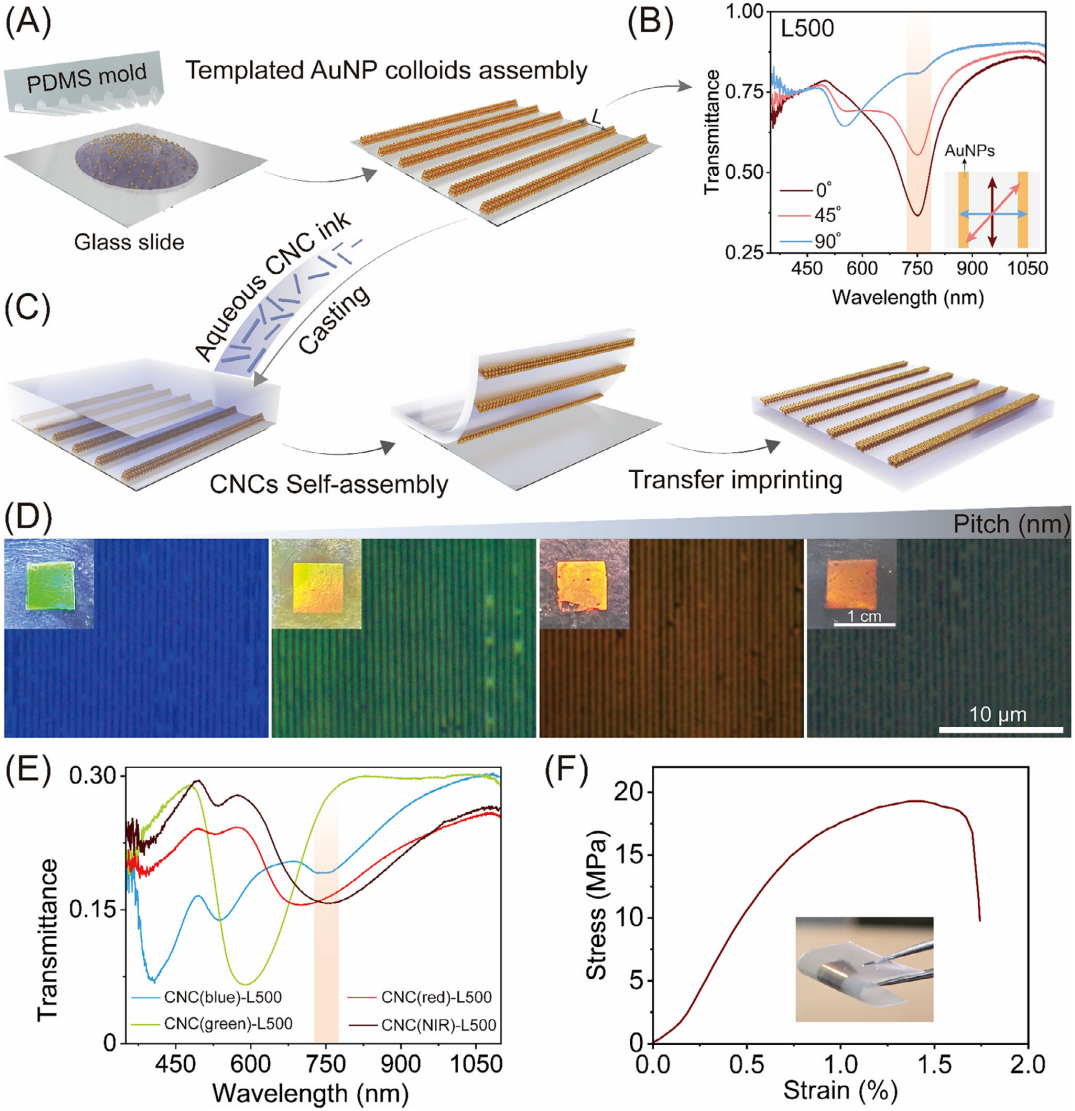
Design, fabrication, and characterization of the plasmonic nanocellulose composites. (A) Schematic illustration of the evaporation‐assisted self‐assembly of AuNPs into an ordered array on a glass substrate. (B) Transmittance spectra of the plasmonic array at linear polarization angles of 0°, 45°, and 90°, displaying polarization‐dependent SLR behavior. The SLR response is highlighted by a light orange rectangle. (C) Illustration of the transfer imprinting process to relocate the plasmonic array from glass onto the surface of CNC film. (D) POM images of the hybrid composite films featuring the periodic AuNP array with a lattice spacing of 500 nm and optically tunable cholesteric bulk matrix. Inset: photographs of the hybrid composites with vivid structural color due to the combination of helical matrix and plasmonic gratings. (E) UV–vis–NIR transmittance spectra of the CNC(x)‐L500 hybrid composites under unpolarized incident light, showing distinct spectral features from bulk cholesteric matrix and surface plasmonic arrays. (F) Stress–strain curve of the CNC(NIR)‐L500, indicating excellent mechanical strength and flexibility of the hybrid film.

To obtain the LD‐active plasmonic nanostructures, linearly assembled plasmonic arrays were prepared through template‐assisted self‐assembly of AuNPs using a polydimethylsiloxane (PDMS) mold with topographically pre‐patterned features (Figure 1A). AuNPs with an average diameter of 30 ± 1.5 nm were synthesized using the cetyltrimethylammonium chloride seed‐mediated growth method and subsequently coated with poly(ethylene glycol) methyl ether thiol (mPEG‐SH, Mw = 2000 g·mol^−1^) to ensure the colloidal stability at high concentrations (Figure S1). The obtained mPEG‐SH functionalized AuNP suspension (2 µL, 50 mm) was then deposited onto a hydrophilic glass slide, and the system was subjected to confined drying by placing the hydrophobic pre‐patterned PDMS stamp on top. As the water evaporated, van der Waals and capillary forces directed the nanoparticles into linear chains that adhered to the template's topography [53]. After removing the PDMS mold, a centimeter‐scale plasmonic grating was successfully assembled onto the glass slide, showing vivid iridescent color due to the grating effect (Figure S2). This template‐assisted self‐assembly approach circumvents conventional high‐cost, cleanroom‐dependent nanofabrication tools (e.g., electron‐beam lithography), offering a colloid‐based and scalable route for producing large‐area plasmonic patterns.

The optical anisotropy of the plasmonic array with a lattice spacing of 500 nm was confirmed by rotating the polarization of incident light from 0° (parallel) to 90° (perpendicular) relative to the AuNP chains orientation (Figure 1B). When the polarization was perpendicular to the grating direction, the spectrum was dominated by the localized surface plasmon resonance (LSPR) of the AuNPs, centered at 540 nm. In contrast, parallel polarization induced a pronounced dip at 750 nm, characteristic of SLR resulting from the collective excitation of longitudinal modes, facilitated by diffractive coupling within the periodic AuNP array [53, 54]. The degree of anisotropy can be quantified using the LD ratio, defined as 2(*A*_⊥_ − *A*_∥_)/(*A*_⊥_ + *A*_∥_), where *A*_⊥_ and *A*_∥_ represent the extinctions at 90° and 0° polarization [30], respectively (Figure S3). When the lattice spacing was 500 nm, we calculated a LD ratio of 0.62 ± 0.04, suggesting the strong LD of the periodic plasmonic array. In addition, the spectral position of SLR can be tuned from visible to near‐infrared (NIR) region by varying the PDMS template spacing from 400 to 600 nm (Figure S4), offering design flexibility for wavelength‐selective optical components. Notably, no chiroptical activity was observed, confirming that the plasmonic grating is intrinsically achiral (Figure S5).

The plasmonic nanocellulose composite films were fabricated by integrating periodic AuNP arrays onto the surface of the cholesteric CNC matrix through the evaporation‐assisted transfer imprinting lithography (Figure 1C). Typically, an aqueous CNC suspension (5 wt.%) was mixed with poly(ethylene glycol) (PEG, 20 kDa) at a fixed mass ratio of 5:2 (CNC‐to‐PEG) and served as the ink. The CNCs used in this study are negatively charged, showing a rodlike morphology with average lengths of 194 ± 54 nm and widths of 9 ± 2 nm (Figure S6). Direct casting of the CNC‐PEG ink onto the pre‐assembled plasmonic array led to partial dissolution and disruption of the AuNP chains (Figure S7). To overcome this issue, an annealing step was introduced to stabilize the AuNP arrays, preserving their optical characteristics and ensuring a successful pattern transfer (Figure S8). After evaporation, birefringent cholesteric composites were formed and peeled off, yielding free‐standing films with periodic AuNP arrays retained at the surface. The seamless integration of this evaporation‐assisted transfer step with the preceding soft‐nanoimprinting colloidal assembly establishes a cost‐effective and scalable pathway for manufacturing large‐area plasmonic‐photonic composites.

The obtained plasmonic nanocellulose composites were labeled as CNC(x)‐L(y), where x represents the structural color of the cholesteric matrix (blue, green, red, or NIR) that controlled by sonicating CNC suspensions prior to evaporation (Table S1), and y denotes the lattice spacing of the plasmonic arrays. Polarized optical microscopy (POM) imaging of the hybrid films revealed periodic parallel stripes and a distinct birefringent CNC matrix, indicating the successful transfer of a linearly assembled AuNP array through evaporation‐induced cholesteric self‐assembly of CNCs (Figure 1D). Macroscopically, under normal incidence inspection, the non‐patterned areas displayed tunable structural colors corresponding to the varying helical pitch, while the patterned regions exhibited intensified iridescence (Figure 1D inset; Figure S9). This can be ascribed to the combined optical effects of the surface plasmonic diffraction grating and the underlying cholesteric bulk matrix, which result in enhanced light scattering and angle‐dependent structural color [49]. To further confirm this optical coupling, we performed UV–vis–NIR spectroscopy using unpolarized light on these hybrid films, displaying spectral features from both the cholesteric matrix and plasmonic arrays (Figure 1E). The hybrid films of CNC(blue)‐L500 and CNC(NIR)‐L500 exhibited a distinct LSPR peak at 540 nm and SLR peak at 750 nm, respectively, separating from their corresponding PBG at 400 and 1300 nm (Figure 1E; Figure S10). In contrast, for the sample of CNC(green)‐L500 and CNC(red)‐L500, the LSPR or SLR peak of linearly assembled AuNP arrays overlap with the PBG of the cholesteric matrix, displaying plasmonic‐photonic coupling at 580 and 700 nm, respectively. The resulting spectra revealed a distinct optical modulation across the visible to NIR range, arising from the custom‐tailored hierarchical structure that integrates tunable cholesteric bulk matrix with surface patterned AuNP arrays. In addition to their optical tunability, these plasmonic composite films exhibited notable mechanical robustness and could be easily disassembled in water, offering a promising route to fabricate sustainable, biodegradable photonic materials (Figure 1F; Figure S11).

**FIGURE 2:**
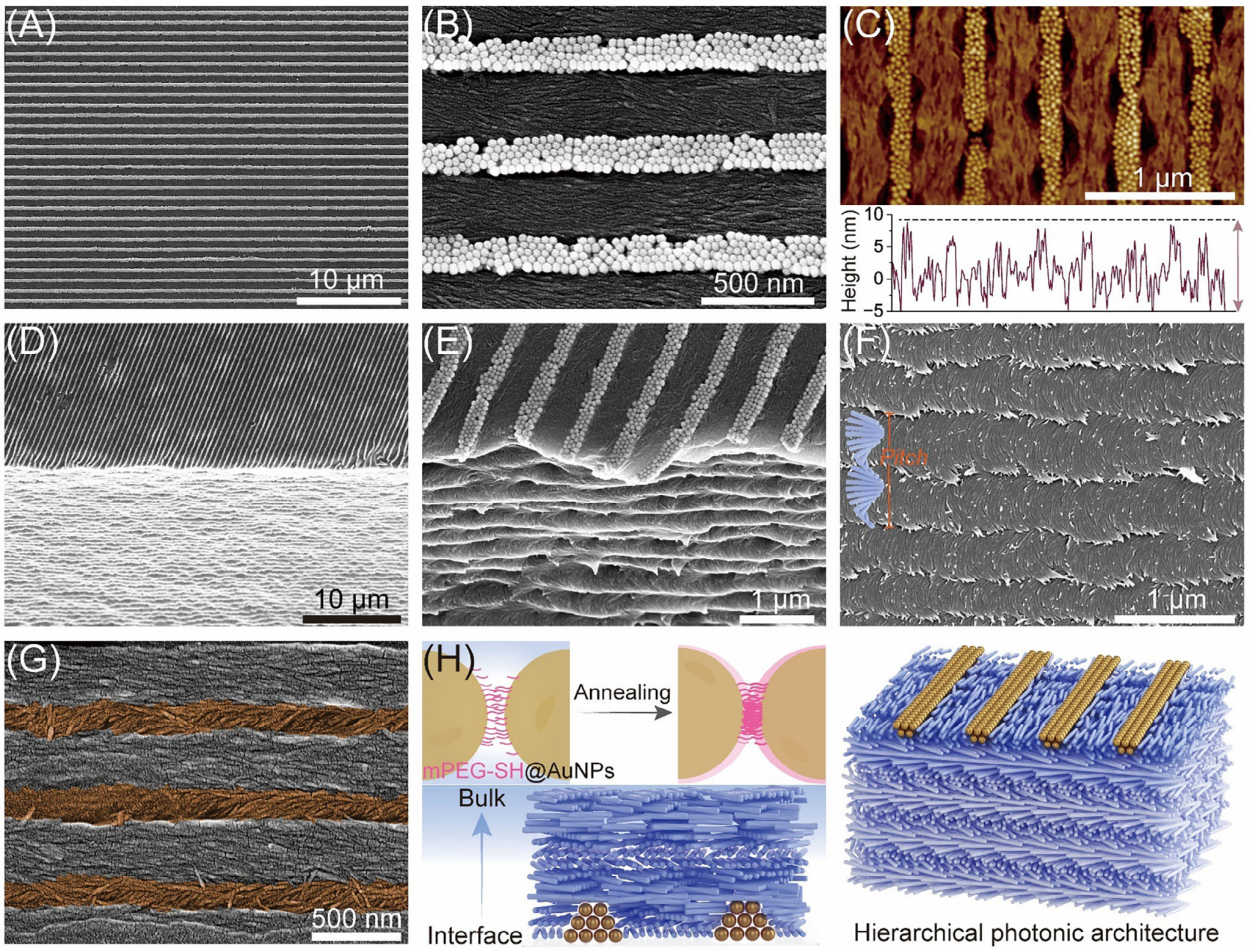
The surface morphology and structure characterization of the plasmonic nanocellulose composites. SEM imaging of the sample of CNC(NIR)‐L500 at low (A) and high‐magnification (B), showing periodically linearly assembled AuNP array with a lattice spacing of 500 nm on the film surface. (C) AFM analysis of the surface topography of AuNP array on the film surface, showing a height of ∼12.5 nm. (D), (E) Side view of the interface between AuNP array and cholesteric CNC bulk phase at different magnifications, revealing the integration of plasmonic grating on the film surface. (F) High‐magnification SEM image of the underlying layered cholesteric matrix, indicating the linearly assembled AuNPs remain intact, without isolated nanoparticles diffusing into the bulk phase. (G) High‐magnification SEM image of the film surface after removal of AuNP chains, displaying the nematic orientation of CNCs between the AuNP array and twisted alignment of CNCs under the AuNP array (highlighted in brown). (H) Schematic illustration of the co‐assembly of CNCs with AuNP array during the transfer imprinting process and the resulting hierarchical photonic architecture.

The surface morphology and inherent structural characteristics of the hybrid composites were examined using scanning electron microscopy (SEM) and atomic force microscopy (AFM). AFM analysis of the plasmonic array on a glass substrate revealed hexagonally packed AuNPs arranged in a linear pattern, with each line measured approximately 89 ± 2 nm in height, corresponding to nearly three layers of nanoparticles (Figure S12). After transfer, the surface of the hybrid composite retained a large‐area micropatterned AuNP chains, closely resembling the pre‐patterned template (Figure 2A,B). This confirms the successful transfer imprinting of the plasmonic array onto the CNC film. Figure 2C presents the AFM surface topography of the resulting hybrid composite film. Compared to the pre‐patterned plasmonic template, the AuNP chains on CNC film surface exhibited a reduced height of 12.5 ± 1.2 nm. This suggests that only the bottommost layer of the original AuNP chains remains exposed, while the upper layers of AuNP assemblies are fully embedded within the CNC matrix. The top surface of the hybrid composite showed a well‐defined AuNP grating, while perpendicular to the surface, we observed a periodic layered structure of CNCs, indicative of preserved cholesteric organization (Figure 2D). A closer inspection of the film cross‐section, we found that the embedded AuNP assemblies remain intact, with no discrete nanoparticles observed in the interfacial region or within the cholesteric bulk (Figure 2E,F; Figure S13), indicating strong structural integrity and minimal nanoparticle diffusion during the transfer imprinting process.

To further investigate how the CNCs co‐assembled in the vicinity of the AuNP array, the AuNP chains were selectively removed (see morphological characterization). As shown in Figure 2G, the topmost rodlike CNC nanoparticles located between the linear AuNP chains exhibited planar anchoring at the surface and were oriented parallel to the axis of the array. This alignment is driven by capillary forces generated by neighboring AuNP chains during evaporation, guiding CNCs into a nematic arrangement between the AuNP chains. Interestingly, compared to the upper layer, a slight helical rotation was observed as the CNC layers followed the groove contours, suggesting the onset of cholesteric organization beneath the patterned surface. This observation highlights the strong interfacial interaction between the surface plasmonic array and the self‐assembled CNCs during the transfer imprinting process. The alignment and deformation of the cholesteric layers near the patterned regions imply that the presence of topographical constraints can guide local CNC orientation without disrupting the overall helical organization. Such interfacial anchoring not only preserves the hierarchical structure but also enhances the optical coupling between the surface plasmonic grating and the underlying cholesteric matrix, offering a custom‐tailored platform to engineer guest‐host optical responses through nanoscale structural design.

Based on the above, Figure 2H illustrates the co‐assembly of CNCs with AuNP array during the transfer imprinting process, along with the resulting hierarchical structure. Before transfer, the pre‐patterned AuNP array is annealed at 100°C to remove residual volatile solvents, allowing neighboring AuNPs to partially reorient and entangle with functionalized mPEG‐SH chains (melting point ∼ 50°C–55°C), resulting in tighter packing. This treatment locks the AuNP array in place and prevents rehydration during subsequent processing. Deposition of the CNC suspension onto the pre‐treated plasmonic array initiates the hierarchical co‐assembly process. During evaporation, the CNCs begin to self‐assemble into a cholesteric liquid crystalline phase, with their alignment influenced by the underlying AuNP array. The spatial confinement and surface topography of the AuNP array induce localized alignment of CNCs at the interface, promoting planar anchoring and guiding the cholesteric axis perpendicular to the substrate. Meanwhile, the capillary effects between neighboring AuNP chains drive the bottommost layer of CNCs into a nematic arrangement. As drying proceeds, the hierarchical order becomes kinetically locked in, resulting in a solid film with a vertically aligned cholesteric matrix and surface‐embedded plasmonic grating. This co‐assembly process yields a multiscale photonic architecture, where the cholesteric ordering propagates into the bulk while the plasmonic array remains partially embedded at the surface.

Building on the above structural analysis, we investigated how the coupling between the linearly assembled AuNP grating and the cholesteric CNC matrix governs the LD and CD of the hybrid films. The pristine cholesteric CNC matrix inherently exhibits a broad positive CD peak at PBG due to the selective reflection of LCP light by its polydomain left‐handed helicoidal structure. To eliminate spectral overlap from this intrinsic PBG signal and unambiguously resolve the plasmonic contribution, we selected the CNC(NIR)‐L500 sample with a thickness of 115 ± 1 µm for detailed LD and CD characterization at the SLR. In this system, the PBG is centered at 1300 nm, spectrally well‐separated from the SLR of the AuNP array at 750 nm (Figure S10).

**FIGURE 3:**
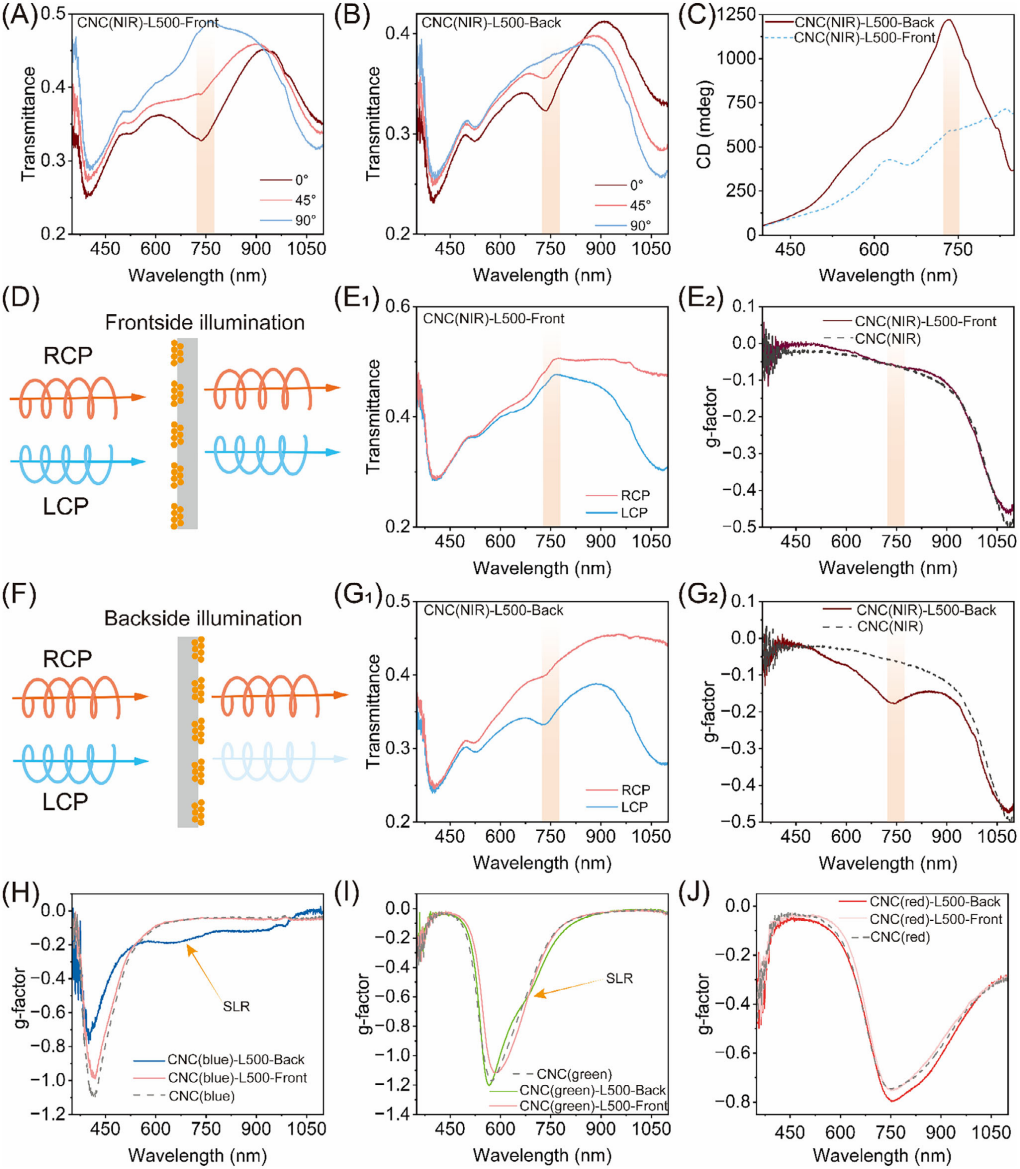
“Front‐and‐back” optical measurements of the plasmonic nanocellulose composites. Transmittance spectra of the CNC(NIR)‐L500 measured at linear polarization angles of 0°, 45°, and 90°, from the frontside (A) and backside (B) illumination, displaying polarization‐dependent SLR behavior. (C) “Front‐and‐back” CD spectra of the CNC(NIR)‐L500, indicative of LB behavior. The SLR response is highlighted by a light orange rectangle. Schematics of frontside LCP/RCP illuminations (D) and the corresponding transmission spectra for CNC(NIR)‐L500 (E_1_), with the calculated g‐factor profile (E_2_). Schematics of backside LCP/RCP illuminations (F) and the corresponding transmission spectra for CNC(NIR)‐L500 (G_1_), as well as the associated g‐factor profile (G_2_). “Front‐and‐back” chiroptical responses of the plasmonic nanocellulose composites with varied structural color in cholesteric matrix with blue (H), green (I), and red (J) appearance, showing tunable plasmonic‐photonic coupling.

To verify the role of the plasmonic array as a polarizing element, we first performed a series of “front‐and‐back” transmittance measurements using linearly polarized light. Under frontside illumination (CNC(NIR)‐L500‐Front), where light first encounters the AuNP surface grating, the film exhibited a polarization‐dependent SLR with a notable LD ratio of 0.22 ± 0.04 (Figure 3A). Conversely, backside illumination of the sample, where light traversed the cholesteric layer before reaching the AuNP array (CNC(NIR)‐L500‐Back), resulted in a reduced LD ratio of 0.07 ± 0.01 at the SLR (Figure 3B). These results are in sharp contrast to the negligible LD of the pure CNC film at 750 nm (Figure S14), confirming that the plasmonic grating serves as the primary LD‐active element in the hybrid film. Compared to the LD ratio (0.62 ± 0.04) of the pre‐assembled AuNP array on glass, the reduced LD ratios in the plasmonic nanocellulose films can be attributed to diffuse scattering and depolarization of the cholesteric CNC matrix [55, 56]. Specifically, the intense scattering from the abundant domain boundaries disrupts the long‐range phase coherence required for collective lattice modes, leading to significant attenuation in the SLR peak intensity. Consequently, the stronger attenuation of the LD ratio under backside illumination aligns with the measurement geometry, where the scattering medium precedes the polarizing element, thereby disrupting the polarization state of the incident light. Despite the weaker LD detected in the backside configuration, this geometry produced a massive CD signal of 1217 ± 51 mdeg at the SLR, far exceeding its frontside counterpart (Figure 3C). The differences in CD magnitude between frontside and backside measurements suggest the giant chiroptical signal arises not from the intrinsic chirality of the cholesteric CNC substrate, but from the effective coupling between the birefringent CNC matrix and the plasmonic grating. Notably, this plasmonic CD magnitude under backside measurement surpasses all previously reported values for plasmonic‐biopolymer composites (Table S2).

To further elucidate the resulting chiral plasmonic optical response, we measured the transmitted LCP (*T_LCP_*) and RCP (*T_RCP_*) spectra through “front‐and‐back” optical tests and calculated the optical dissymmetry factor using gfactor = 2(*T_LCP_* − *T_RCP_*)/(*T_LCP_* + *T_RCP_*). Unlike conventional CD spectroscopy used above, which measures only differential absorption, this transmission‐based g‐factor accounts for dominant scattering and reflection contributions in both the achiral plasmonic arrays and the cholesteric CNC structure [57]. When light illuminated and pass through the patterned side of the plasmonic nanocellulose composite (CNC(NIR)‐L500‐Front), the measured LCP and RCP spectra were nearly identical without a SLR feature (Figure 3D, E_1_), yielding a g‐factor of −0.05 that is similar to the pure CNC film (Figure 3E_2_; Figure S14). This observation serves as a vital control: it aligns that the achiral plasmonic grating, when interacting with CPL light before the chiral matrix, is incapable of inducing CD, effectively acting merely as a passive layer. By contrast, the backside illumination (CNC(NIR)‐L500‐Back) displayed a strong SLR dip in the LCP spectrum at 750 nm, indicative of an effective chiral coupling between LCP light and the plasmonic grating (Figure 3F,G_1_). This produced an enhanced g‐factor of −0.19 ± 0.02 (Figure 3G_2_), demonstrating that the plasmonic grating acts as a LD‐active layer significantly amplifies the chiral character imparted by the preceding CNC matrix. We further examined the impact of the thickness of the cholesteric CNC matrix and observed that the g‐factor remained consistent even as the thickness of the CNC(NIR)‐L500 film was increased from 115 to 324 µm (Figure S15). Furthermore, the plasmonic CD signal of SLR can be tuned by varying the lattice spacing from 400 to 600 nm, displaying comparable g‐factors at their respective SLR wavelengths under backside illumination (Figure S16). These results demonstrate that the plasmonic nanocellulose composites exhibit strong, tunable plasmonic CD, with the magnitude and spectral response critically dependent on the stacking order of the cholesteric CNC matrix and the AuNP array relative to the incident light.

Beyond the geometric tuning of the plasmonic lattice described above, the cholesteric CNC matrix offers a complementary degree of freedom to manipulate the light‐matter interaction. To engineer the plasmonic‐photonic chiroptical responses, we tuned the PBG of the cholesteric CNC matrix across the range of 400–800 nm while maintaining the AuNP lattice spacing of 500 nm to place SLR at 750 nm. When the PBG is far away from the SLR of the surface AuNP array, the sample produced a CD signature without plasmonic contribution under frontside illumination (CNC(blue)‐L500‐Front), similar to the pure CNC counterparts. However, when the sample was illuminated from the backside (CNC(blue)‐L500‐Back), the sample developed a chiroptical SLR response with a g‐factor of −0.19, coexisting with a g‐factor of −0.78 from the cholesteric CNC matrix (Figure 3H; Figure S17). As the PBG approached the SLR region, establishing a partial spectral overlap, a transition in the optical response was observed. Under backside illumination, a weak chiral plasmonic CD feature emerged at 710 nm in the spectrum of the CNC(blue)‐L500 film, which remained spectrally distinct from the primary PBG peak at 580 nm (Figure 3I; Figure S18). Notably, when the PBG was tuned to spectrally overlap with the SLR, the plasmonic and photonic responses merged into a single broad feature in the spectrum of the CNC(red)‐L500 film (Figure 3J; Figure S19). This spectral coalescence is indicative of strong plasmonic‐photonic chiroptical coupling. These results establish the plasmonic nanocellulose composite as a versatile chiral platform, enabling programmable coupling or decoupling of intrinsic cholesteric chirality and extrinsic plasmonic chirality to engineer tailored chiroptical functionalities from the visible to the near‐infrared range.

**FIGURE 4:**
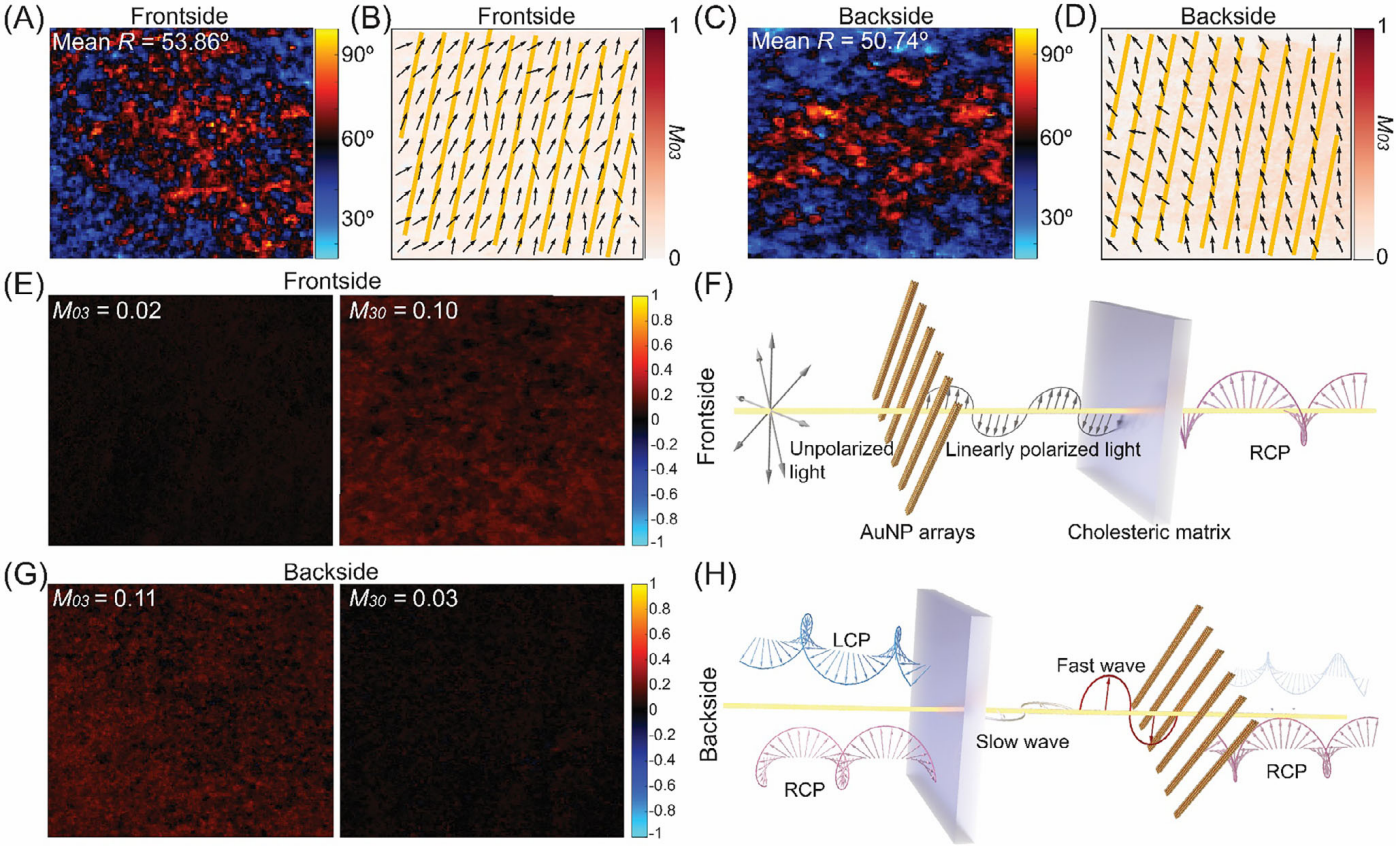
MM polarimetry measurements of the hybrid plasmonic nanocellulose film. Under frontside illumination, the calculated retardance map for the CNC(blue)‐L400 (A) and the corresponding fast‐axis (black arrows) of the birefringent CNC matrix (B), showing a finite tilt relative to the LD axis of the AuNP chains (yellow line). Under backside illumination, the calculated retardance map for the CNC(blue)‐L400 (C) and the corresponding fast‐axis of the birefringent CNC matrix (D), showing unidirectionally aligned with a finite tilt relative to the LD axis of the AuNP array. (E) CD (*M*_03_) and circular polarizance (*M*_30_) of the CNC(blue)‐L400 under frontside illumination. (F) Schematic illustration of the corresponding LD‐LB optical configuration under frontside illumination. (G) CD (*M*_03_) and circular polarizance (*M*_30_) of the CNC(blue)‐L400 under backside illumination. (H) Schematic illustration of the reversed LB‐LD stacking order under backside illumination.

To elucidate the origin of the plasmonic chiroptical response, we performed MM polarimetry, which enables a comprehensive characterization of LD, CD (*M*_03_), circular polarizance (*M*_30_) and linear retardance (*R*) of the hybrid films. The sample CNC(Blue)‐L400 was selected for MM analysis, as its SLR at 633 nm falls within the operating range of the MM polarimeter. The pristine CNC(Blue) film showed negligible LD and CD but displayed a spatially heterogeneous retardance distribution with a mean retardance of *R* = 28.7° at 633 nm (Figure S20). This indicates that the cholesteric CNC matrix can act as a retarder, introducing a phase shift between orthogonal polarization components of the incident light. Retardance vectors retrieved from MM data further revealed a randomly oriented fast‐axis distribution within the pristine cholesteric CNC matrix (Figure S20). In contrast, deposition of AuNP array introduced notable linear diattenuation (D) and increased linear retardancy under both frontside (*D* = 0.30, *R* = 53.86°) and backside (*D* = 0.24, *R* = 50.74°) illumination (Figure 4A,C; Figures S21 and S22). MM vector analysis further revealed that the fast axis of the birefringent CNC matrix was more unidirectionally aligned under backside illumination than frontside illumination, while in both cases, a finite tilt existed between LB axis of CNC matrix and the LD axis of the AuNP array (Figure 4B,D). These results highlight a structured coexistence of LD from the plasmonic array and phase retardance from the cholesteric CNC matrix, together shaping the observed plasmonic‐photonic chiroptical response.

Based on the above, we further examined how the interplay between the birefringence and LD shapes the chiroptical response of the hybrid films. Under frontside illumination, CNC(blue)‐L400‐Front exhibited a negligible CD (*M*_03_ = 0.02) but a pronounced circular polarizance (*M*_30_ = 0.1 and Figure 4E). Given that the values of *M*_03_ and *M*_30_ range from –1 to +1, representing preferential transmission of LCP and RCP light, respectively. This indicates that the CNC(blue)‐L400‐Front can act as a circular polarizer to convert incident unpolarized light into predominantly RCP state. This behavior can be attributed to the sequential LD‐LB configuration, in which the AuNP grating first generates linearly polarized light that is subsequently converted to circular polarization via the retardance from the birefringent cholesteric CNC matrix (Figure 4F). In contrast, the CNC(blue)‐L400‐Back exhibited an enhanced CD (*M*_03_ = 0.11) accompanied by a weak circular polarizance (*M*_30_ = 0.03), indicating preferential attenuation of transmitted LCP (Figure 4G). In this scenario, the CD signals arise from the LB‐LD stacking sequence, where the incident light is first split into fast and slow polarization components by the birefringent CNC matrix, which then interferes after transmission through the LD‐active AuNP array that acts as an imperfect polarizer, thus generating strong plasmonic CD (Figure 4H; Figure S23). Taken together, these results reveal an inversion of chiroptical response upon reversing the illumination direction, manifesting as antisymmetric CD behavior. Such directional‐dependent plasmonic CD, uncommon in conventional chiral materials, provides promising opportunities for bidirectional photonic devices and mirrored optical architectures where selective control over opposite chiral states is essential [58].

**FIGURE 5:**
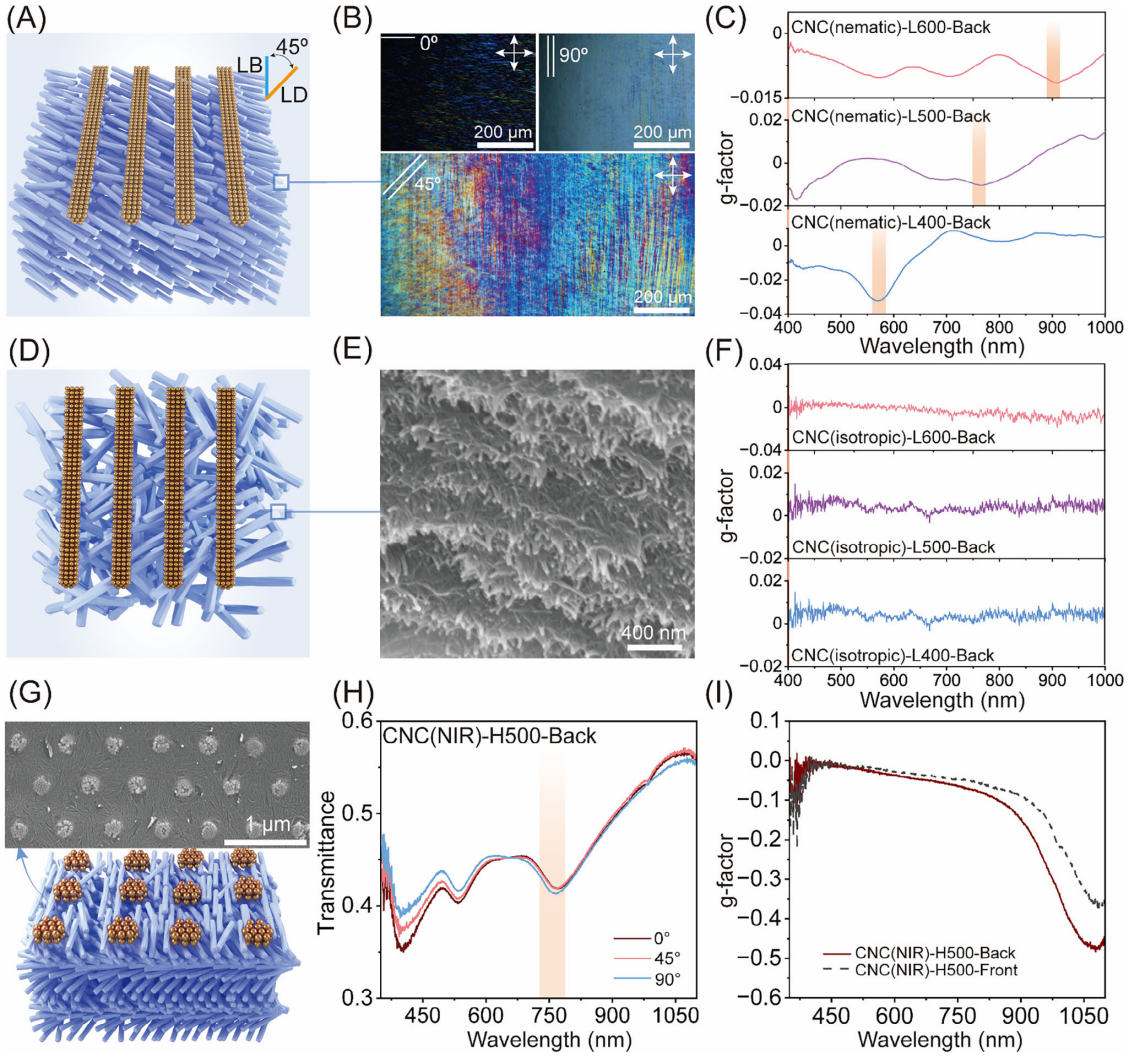
Tunning CNC self‐assembly and plasmonic lattice geometry to engineer chiroptical properties. (A) Schematic illustration of the plasmonic nematic CNC composite film, featuring unidirectionally aligned CNCs in the bulk matrix and a plasmonic grating oriented at 45° with respect to the nematic axis. (B) POM images showing strong birefringence of the nematic CNC matrix when the incident light polarization is oriented at 45° compared to 0° and 90°. (C) Chiroptical responses of the plasmonic nematic composite films with the lattice spacing varying from 400, 500 to 600 nm. The tilt angle between the orientation of CNC matrix and the plasmonic grating is fixed at 45°. The SLR response is highlighted by a light orange rectangle. (D) Schematic illustration of the plasmonic CNC(isotropic) composite film with a disordered structure in CNC matrix integrated with an AuNPs grating. (E) Highly magnified SEM image of the isotropic CNC matrix. (F) The corresponding g‐factor profiles of the plasmonic nanocellulose composite films with isotropic CNC matrix and varied lattice spacing from 400, 500 to 600 nm of AuNP array. (G) Schematic illustration and SEM image of the plasmonic nanocellulose composite featuring a square lattice plasmonic array integrated with the cholesteric CNC matrix. (H) Transmittance spectra of the CNC(NIR)‐H500‐Back under rotating linear polarization, confirming the polarization‐independent response and absence of LD. (I) The corresponding g‐factor profile of the CNC(NIR)‐H500 film, confirming the absence of plasmonic chiroptical activity.

To further validate the LB‐LD interference mechanism, we considered the MM relation predicting that CD arises from the combination of LB, LD, and the tilt angle (α) between their optical axes, following *CD*∝*LB* × *LD* × *cos*(2α − 90°) [58, 59, 60]. To disentangle these contributions, we systematically varied the CNC organization and geometry of plasmonic nanostructures in the plasmonic nanocellulose composites. First, we replaced the cholesteric CNC substrate with a nematic CNC matrix, which retains LB but eliminates intrinsic chirality. CNCs were aligned into unidirectional nematic order in the wet state by blade coating onto pre‐assembled plasmonic gratings, with the tilt angle α set to either 0° or 45° (Figure 5A; Figure S24). Upon drying, POM imaging showed maximum brightness when the average CNC orientation was at 45° relative to the crossed polarizers, confirming the preservation of nematic orientation in the hybrid film (Figure 5B). Under backside illumination, distinct SLR‐induced chiroptical signals were observed in CNC(nematic)‐L400, L500, and L600 films when α = 45° (Figure 5C), whereas parallel alignment (α = 0°) produced no measurable chiroptical response (g − factor ≈ 0, Figure S24). Next, the CNC orientation was disrupted by adding sodium chloride, yielding isotropic dispersions that were cast onto pre‐assembled plasmonic AuNP arrays (Figure 5D,E). The resulting CNC(isotropic)‐L400, L500, and L600 films lacked birefringence and showed no chiroptical activity under backside illumination (Figure 5F; Figure S25). These results highlight that macroscopic birefringence of the CNC matrix and a finite tilt angle between the LB and LD axes are essential for the emergence of plasmonic CD.

Finally, to assess the role of LD, we preserved the cholesteric CNC matrix with its PBG in the NIR region while replacing the linearly aligned AuNP array with a square‐lattice AuNP array (Figure 5G). The 2D lattice array (lattice spacing 500 nm) produced polarization‐independent SLR at 750 nm and a zero g‐factor, reflecting the symmetry of AuNP clusters along both orthogonal axes (Figure S26). Transferring imprinting of the square lattice onto the cholesteric CNC matrix yielded the CNC(NIR)‐H500 composite, which displayed polarization‐insensitive SLR at 750 nm, confirming the absence of LD (Figure 5H). Correspondingly, no SLR‐associated chiroptical signal was observed, despite the presence of CNC‐induced birefringence (Figure 5I). These results establish that the LD‐active plasmonic gratings are indispensable for generating plasmonic CD. We therefore confirm that the observed CD signals at the SLR originate exclusively from cooperative LB‐LD interference, requiring birefringence in the CNC matrix, anisotropic plasmonic arrays, and a non‐zero tilt angle α, consistent with the MM analysis.

## 3 Discussion

We report a plasmonic nanocellulose composite that exhibits tunable plasmonic‐photonic coupling and custom‐tailored chiroptical response by integrating a LD‐active plasmonic grating with a LB‐active cholesteric CNC matrix. The hybrid composites are fabricated by casting aqueous CNC colloids onto pre‐assembled AuNP arrays followed by drying and peeling off from the template, resulting in nanoscale cholesteric organization of CNCs and microscale periodic surface patterned AuNP metasurface. Morphological and structural analyses validate the formation of multiscale photonic architectures in an integrated platform, which highlights the unique capability of biobased self‐assembling substrates to construct scalable, centimeter‐scale plasmonic metasurfaces. Owing to the Janus surface structure, frontside AuNP plasmonic grating arrays and backside smooth chiral bulk phase, the hybrid films display a distinctive illumination‐dependent plasmonic chiroptical response that arises from optical symmetry breaking. Besides, the resulting chiroptical activity can also be tuned by manipulating both the SLR of the nanoparticle arrays and the PBG of the cholesteric CNC matrix. MM polarimetry measurement reveals that the plasmonic CD originates from the cooperative LB‐LD interplay, in which the cholesteric CNC layer acts as a phase retarder and the plasmonic grating functions as a linear polarizer. Our work demonstrates that leveraging this LB‐LD coupling mechanism effectively overcomes the intrinsic limitations of traditional plasmonic‐biopolymer chiral systems that are restricted by the complex interplay of geometric and resonant parameters. Overall, these findings establish a clear design principle for tailoring chiroptical responses and position plasmonic nanocellulose composites as a sustainable, scalable platform for next‐generation chiral photonics, with potential applications in polarization‐encoded optical communication, quantum photonic circuits, and ultrasensitive chiral biosensing.

## 4 Experimental Section

### 4.1 Chemicals and Materials

Hexadecyltrimethylammonium chloride (CTAC, 25 wt.% in water), poly(ethylene glycol) methyl ether thiol (mPEG‐SH, MW 2000 g⋅mol^−1^), Hydrogen tetrachloroaurate trihydrate (HAuCl_4_·3H_2_O, ≥ 99.9%), trichloro(1H,1H,2H,2H‐perfluorooctyl)silane (97%), 1,3,5,7‐tetramethyl‐1,3,5,7‐tetravinyl‐cyclotetrasiloxane, platinum catalyst, hydroxysiloxane and poly(ethylene glycol) (PEG, 20 kDa) were purchased from Sigma–Aldrich. L‐ascorbic acid (AA, ≥ 99%) was purchased from Alfa Aesar. Sodium borohydride (NaBH_4_, 99%) and sodium hypochlorite (NaClO, 10‐15% active chlorine) were purchased from Acros Organics. OrmoStamp was purchased from Micro Resist Technology. Acetone (≥ 99.6%), toluene, 2‐propanol (≥ 99.9%), and ethanol (≥ 96%) were purchased from Labbox. Polydimethylsiloxane (PDMS, Sylgard 184) was purchased from Dow Corning (Michigan, USA). All chemicals were used as received. Milli‐Q water was used in all experiments. Glass slides (Labbox Spain, 18×18 mm^2^) were sonicated with acetone and isopropanol for 10 min each and irradiated by UV‐ozone for 10 min. The other glasswares were washed with aqua regia (3:1 HCl:HNO_3_) and Milli‐Q water, and dried before use. Sulfated cellulose nanocrystal gel (10.5 wt.%) was purchased from the U.S. Forest Products Laboratory at the University of Maine.

### 4.2 Preparation of PDMS Molds

Silicon masters with periodic linear topography with different lattice spacings were fabricated by electron beam lithography and subsequently used for hard‐PDMS (h‐PDMS) stamp fabrication. To prevent sticking during replication, the silicon master underwent silanization with 8 µL of trichloro(1H,1H,2H,2H‐perfluorooctyl)silane in vacuum for 30 min. To create h‐PDMS stamps, a mixture of vinyl‐methyl‐siloxane, tetramethyl‐1,3,5,7‐tetravinyl‐cyclotetrasiloxane, a platinum catalyst, hydroxysiloxane, and toluene was used. The mixture was cast onto the silicon master and allowed to rest for 1 h to evaporate the toluene. Subsequently, the system underwent curing at 60°C for 1 h. In parallel, soft PDMS was prepared by mixing the monomer and curing agent in a 10:1 ratio, degassed under vacuum for 2 h. To create mechanical support for the h‐PDMS layer, the soft PDMS mixture was applied over the cured h‐PDMS surface. Followed a 30‐min vacuum treatment, the entire setup was cured at 100°C for 2 h. Finally, PDMS stamps with lattice spacings of 400, 500, and 600 nm were individually separated from the silicon master and trimmed to the required dimensions. A constant depth of 390 nm was for all templates, and stripe widths of 194 nm (for L400), 263 nm (for L500), and 299 nm (for L600). The PDMS stripe width determines the final array's periodicity. The height and density of the gold nanoparticles in the groove were primarily controlled by the AuNP suspension concentration. Therefore, a gold suspension with a concentration of 50 mm was utilized to ensure sufficient filling to achieve high‐quality resonance.

### 4.3 Synthesis of Gold Nanospheres

Monodisperse AuNPs with a diameter of 30 nm were synthesized using a seed‐mediated growth method, as previously reported [61]. Seed A was prepared by adding 50 µL of 50 mm HAuCl_4_ solution and then 200 µL of 20 mm NaBH_4_ solution to 5 mL of 0.1 m CTAC solution under vigorous stirring for 5 min. The resulting mixture was then diluted to 50 mL with 0.1 m CTAC solution. Subsequently, 900 µL of the diluted seed A solution was added to 10 mL of 25 mm CTAC solution, followed by 40 µL of 10 mg/mL AA solution and 50 µL of 50 mm HAuCl_4_ solution. The mixture was left at room temperature for 10 min to obtain 10 nm AuNPs, denoted as seed B. To synthesize 30 nm gold nanospheres, 3.5 mL of seed B was added to 100 mL of 25 mm CTAC solution, followed by the addition of 250 µL of 0.1 m AA solution and 250 µL of 50 mm HAuCl_4_ solution. The mixture was left for nanoparticles growth at room temperature for 1 h. To improve sphericity, an additional etching step was performed by heating the suspension at 30°C with the addition of 100 µL NaClO solution (1–1.5 wt.%) and 100 µL of 50 mm HAuCl_4_. Finally, 50 µL of mPEG‐SH solution (1 mg/mL) was added to 100 mL of the AuNP suspension, followed by overnight stirring at room temperature, yielding mPEG‐SH functionalized AuNPs.

### 4.4 Preparation of Plasmonic Arrays on Glass Substrates

A 100 mL AuNP suspension was concentrated by centrifugation to a 10 µL stock suspension. In parallel, 6 mL of deionized water, 4 mL of ethanol, and 20 µL of 25 mm CTAC were mixed to form a dispersion solvent. Then, 1 µL of the highly concentrated AuNP suspension was mixed with 9 µL of the dispersion solution. A 2 µL aliquot of the resulting mixture (50 mm) was deposited onto a patterned PDMS mold, and a coverslip was gently placed on top. The system was left undisturbed for 4 h to allow the self‐assembly of AuNPs into linear chains. Afterward, the PDMS mold was carefully removed, leaving behind a well‐defined AuNP array on the glass substrate. To stabilize the AuNP array for subsequent transfer imprinting, the sample was kept at 100°C for around 12 h. The periodical lattice spacing between adjacent nanoparticle chains was determined by the lattice parameter of the PDMS mold.

### 4.5 Preparation of Plasmonic Nanocellulose Composites with Integrated Surface Plasmonic Arrays and Cholesteric Matrix

Suspensions of CNCs (5 wt.%) were treated by tip sonication at an amplitude 60% for various durations (1, 5, 10, and 20 min) in an ice bath. Then, 0.1 g of PEG (20 kDa) powder was dissolved in 5 g of CNC suspension by magnetic stirring at room temperature for 30 min. For film fabrication, 5 g of CNC/PEG dispersion was poured onto a pre‐assembled plasmonic grating and allowed to evaporate in a petri dish over 24 h. After drying, the self‐standing film was gently peeled off from the glass substrate, with one surface patterned by AuNP array. To obtain hybrid films with increased thickness, the initial mass of the suspension was varied from 5, 7.5 to 10 g. The resulting film thicknesses were measured using a stylus profiler (Alpha‐Step D‐500, KLA Corporation, USA). The measured thicknesses were 115 ± 1, 200 ± 1, and 324 ± 2 µm for initial suspension masses of 5, 7.5, and 10 g, respectively.

### 4.6 Preparation of Plasmonic Nanocellulose Composites with Integrated Surface Plasmonic Grating and Nematic Matrix

For the fabrication of nematic CNC films, an automatic coater (ZAA 2300, Zehntner) facilitated the distribution of the CNC suspension (8 wt.%) onto glass microscope slides through a blade coating technique. The blade evenly distributed the dispersion across the pre‐prepared plasmonic array, simultaneously removing any surplus from the lower edge of the slide. Film thickness was controlled by adjusting micrometer gauges on the coater, with wet thicknesses ranging from 100 to 400 µm. The coating speed was calibrated to ensure a consistent shear rate, defined by *V*/*d*(*s* ^−1^) where *V* is the velocity of the blade (mm/s), *d* is the gap of the blade (µm). To ensure retaining the nematic phase after the coating process, a shear rate of 100 s^−1^ was implemented for all films [62]. After drying at room temperature, the plasmonic nematic CNC films were gently peeled off from the glass substrate.

### 4.7 Preparation of Plasmonic Nanocellulose Composites with Integrated Surface Plasmonic Grating and Disordered CNC Matrix

A CNC suspension (5 wt.%) sonicated for 10 min was mixed with 0.3 g PEG under stirring for 2 h. Then, 600 µL of sodium chloride solution (10 mg/mL) was added into CNC/PEG mixture to prevent the formation of cholesteric organization. The resulting mixture was poured onto the plasmonic arrays on glass substrates. After drying 24 h, transparent films with one surface patterned by plasmonic arrays were obtained.

### 4.8 Preparation of Plasmonic Nanocellulose Composites with Integrated Plasmonic Square Lattice Array and Cholesteric Matrix

A plasmonic square lattice array was produced by using a pre‐patterned PDMS mold featuring a square lattice topography with a periodicity of 500 nm. The resulting plasmonic square lattice template was subjected to the same annealing treatment and transferred onto the surface of the cholesteric CNC film using the same transfer imprinting approach described above.

## Author Contributions

G.C. and H.T. conceived the idea. H.T. contributed to methodology, experiments, data analysis, writing, and funding acquisition. S.J. contributed to experiments, data analysis, and writing. G.C. contributed to data analysis, writing, and reviewing. X.Y.Q. contributed to experiments and data curation. I.E. and A.L. contributed to MM experiment and analysis. W.Y.X. contributed to writing and reviewing. S.W.D. contributed to data curation. A.M. contributed to funding acquisition, writing, and reviewing. E.K. contributed to funding acquisition, supervision, writing, and reviewing. The manuscript was written through the contributions of all authors. All authors have given approval to the final version of the manuscript.

## Conflicts of Interest

The authors declare no conflicts of interest.

## Supporting information




**Supporting File**: adma72561‐sup‐0001‐SuppMat.pdf.

## Data Availability

The data that support the findings of this study are available from the corresponding author upon reasonable request.
