# Supplementary material for: Engineering Chiroptical Interactions through Integrating Plasmonic Arrays with Cholesteric Nanocellulose
Source: Adv Mater. 2026 Feb 15;38(16):e19964. doi: 10.1002/adma.202519964 (PMC12994304; doi:10.1002/adma.202519964)
Supplement: Supplementary file 1 — Supporting File: adma72561‐sup‐0001‐SuppMat.pdf. [file ADMA-38-e19964-s001.pdf]

Supporting Information

Engineering Chiroptical Interactions through Integrating Plasmonic Arrays with Cholesteric Nanocellulose

Han Tao[||], *Sunghwan Jo*[||], *Guang Chu*[||, *], *Xiaoyu Qi, Irene Estévez, Angel Lizana, Wenyang Xu, Shengwei Deng, Agustin Mihi*, Eero Kontturi**

H. Tao, E. Kontturi
Department of Bioproducts and Biosystems, Aalto University School of Chemical Engineering, Vuorimiehentie 1, 02510 Espoo, Finland
S. Jo, X. Qi, A. Mihi
Institute of Materials Science of Barcelona ICMAB-CSIC; Campus UAB, Bellaterra, Spain
G. Chu
School of Chemistry and Chemical Engineering, Southeast University, Nanjing 211189, China
I. Estévez, A. Lizana
Grup d'Òptica, Departament de Física, Universitat Autònoma de Barcelona UAB, 08193 Bellaterra, Spain
W. Xu
Max Planck Institute of Colloids and Interfaces, Science Park Golm, 14476 Potsdam, Germany
S. Deng
College of Chemical Engineering, Zhejiang University of Technology, Hangzhou 310014, China

E-mail: chuguang88@gmail.com, amihi@icmab.es, eero.kontturi@aalto.fi
[||]These authors contributed equally to this work

Characterization

Morphological characterization

POM images were acquired using an Olympus BX53-P microscope (Olympus, Japan) equipped with a pair of polarizers in a perpendicular arrangement. A Zeiss Ultra Plus high-resolution SEM was used to characterize the nanostructure of plasmonic arrays on glass substrates and plasmonic nanocellulose composite films. To visualize the interfacial region and helicoidal structure within the bulk, cross sections of the hybrid films were prepared by fracturing samples after immersion in liquid nitrogen for 2 min. To selectively remove AuNP array from the surface of cholesteric matrix, the hybrid film was immersed in liquid nitrogen, and the surface was gently scraped with a blade. The surface and cross sections were then coated with a 3 nm conductive platinum layer using a sputter coater to ensure electron conductivity. All SEM measurements were performed at an accelerating voltage of 5 kV with a working distance of 3–5 mm. The pitch values were measured using ImageJ software. Bruker MultiMode 8 AFM was used to characterize the topography of the plasmonic array and hybrid films in ScanAsyst mode. The height of AuNP chains was analysed with NanoScope Analysis 3.0 software. Transmission electron microscopy (TEM) images were scanned by JEOL 1210 TEM instrument (Tokyo, Japan) operating at 120 kV. 200 mesh carbon film Cu grids were purchased from Electron Microscopy Science.

Mechanical properties characterization

The plasmonic nanocellulose composite films were cut into rectangular specimens using a steel mold, with dimensions of 5 cm × 0.8 cm × 0.8 mm. Tensile stress–strain measurements were performed using a Universal Testing Machine (Instron 4204) under controlled humidity conditions.

Transmittance and circular dichroism measurements

Agilent Cary 5000 UV-Vis-NIR Spectrophotometer was employed to measure the optical properties of the plasmonic nanocellulose films under arbitrary polarization.

Transmittance spectra of the hybrid plasmonic-cellulose films under different linear polarization angles and under circularly polarized light (CPL) illumination were obtained in a custom-built optical setup (Scheme S1). All the measurements were conducted under normal incidence. Unpolarized light from a tungsten halogen lamp (HL-2000-HP, Ocean Optics) passed through a Glan-Thompson linear polarizer (GTH10M, Thorlabs) to generate linearly polarized light. The polarization angle was controlled via a motorized rotation mount (K10CR2,

Thorlabs). To generate CPL, vertically linearly polarized light was transmitted through a superachromatic quarter-wave plate (SAQWP05M-700, 325–1100 nm, Thorlabs). By orienting the fast axis of the wave plate at ±45° relative to the linear polarization axis, left-handed and right-handed circularly polarized (LCP and RCP) light were generated. The quarter-wave plate was mounted on a piezoelectric rotation mount (ELL14, Thorlabs). The light was focused onto the sample using a 4× objective (NA = 0.1). The transmitted light was collected by an identical 4× objective and coupled into a fiber-connected spectrophotometer (Ocean Insight; QEPro-FL for 400–1100 nm or NIR-Quest for 1100 – 1800 nm). This optical setup was operated by automated LabVIEW code.

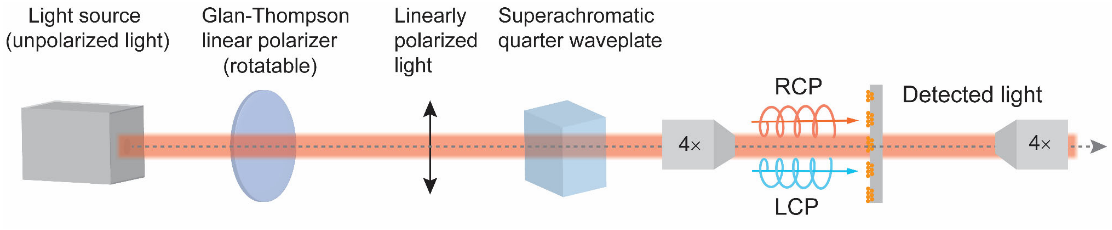

Scheme S1. Illustration of experimental set-up used for characterizing the transmittance and circular dichroism.

Chiroptical properties of the hybrid films were characterized using two different spectroscopic techniques. CD based on the CPL absorption of the hybrid films was measured by a CD spectrometer JASCO J-1500-150ST, which is defined as:

$$CD = a_{LCP} - a_{RCP} \quad (1)$$

Where $a_{LCP}$and a_{RCP} are the absorption for LCP and RCP light, respectively. In this method, the reflection and scattering processes are considered as negligible. Another useful quantity related to the CD is the so-called dissymmetry factor or g-factor, introduced by Tang and Cohen in terms of enantioselectivity with the following expression [1]:

$$\text{g-factor} = 2(a_{LCP} - a_{RCP})/(a_{LCP} + a_{RCP}) \quad (2)$$

This magnitude considers adsorption as the sole source of differential transmittance. In the hybrid plasmonic-photonic systems, however, the diffusive CNC matrix strongly scatters LCP, leading to an underestimation of the differences between LCP and RCP in the transmittance spectra. When CPL is generated by passing linearly polarized light through a polarizer at ±45° relative to the fast axis of a quarter-wave plate and then directed onto the composite films, both absorption and scattering must be considered. Scattering that redirects light out of the propagation axis can also produce differences in the transmitted signals. Thus, even if one circular polarization is scattered rather than absorbed, a differential transmittance is still detected in the forward direction and can be interpreted as CD. For this reason, we redefine

CD and the dissymmetry factor to include scattering effects, following earlier work on periodic arrays of chiral scatterers, where normalization to unpolarized transmittance is applied for the dissymmetry factor [2]. In this study, the transmittance measurements are expressed accordingly:

$$\text{g-factor} = 2(T_{LCP} - T_{RCP})/\,(T_{LCP} + T_{RCP}) \tag{3}$$

Decompositions of Mueller matrix (MM)

The MM data were collected by measuring transmittance under six different polarization states: four linearly polarized states (0°, 90°, +45°, and −45°) and two circularly polarized states (LCP and RCP) at a certain wavelength. These measurements enabled the determination of all 16 MM elements that describe the polarization response of the sample.

The Mueller matrix is a 4×4 matrix based on the Mueller-Stokes formalism. The Mueller-Stokes formalism stands out due to two key advantages: it relies on measurable intensity data, making it experimentally practical, and it can effectively handle depolarized light or depolarizing systems. The Mueller-Stokes formalism represents the polarimetric state of light using a 4×1 matrix comprising the four Stokes parameters: S0, S1, S2, and S3. These parameters correspond, respectively, to the total intensity of the beam, the degree of linear polarization along the horizontal/vertical (x/y) and ± 45° orientations, and the degree of circular polarization (left/right handedness). To characterize light-matter interactions within this framework, the MM is employed. The MM is defined mathematically as follows:

$$\vec{S}_{out} = M \cdot \vec{S}_{in} = \begin{pmatrix} m_{00} & m_{01} & m_{02} & m_{03} \\ m_{10} & m_{11} & m_{12} & m_{13} \\ m_{20} & m_{21} & m_{22} & m_{23} \\ m_{30} & m_{31} & m_{32} & m_{33} \end{pmatrix} \vec{S}_{in} \tag{1}$$

The elements of the MM contain information about the polarimetric characteristics of a sample. While certain elements can be directly linked to physical properties, others require mathematical processing, such as matrix decomposition, to extract meaningful insights. The MM is rewritten as Eq. (2) in the simple form for the direct interpretation.

$$M = \begin{pmatrix} m_{00} & \vec{D} \\ \vec{P} & m_R \end{pmatrix} \tag{2}$$

where m_{00}, $\vec{D}$ and $\vec{P}$ represent the total intensity of the light, the Diattenuation vector ($\vec{D}$), and the Polarizance vector ($\vec{P}$), respectively.

The determination of optical axis of the LB of cholesteric CNC matrix

In the Mueller matrix formalism, the lower-right 3×3 submatrix m_R which called retarder matrix, excluding the intensity element m_{00} and the diattenuation and polarizance vectors $\vec{D}$

and $\vec{P}$, is mathematically a proper orthogonal matrix that performs a three-dimensional rotation on the Stokes vector components in the Poincaré sphere coordinates, namely (Q = S1, U = S2, V = S3). Physically, the angle of this rotation corresponds to the phase retardance between the fast and slow optical axes of the medium. Therefore, the retardance R represents the magnitude of this rotation and can be expressed in terms of the rotation angle associated with mR, as shown in Eq. (3) [3].

$$R = \left|\vec{\mathrm{R}}\right| = \cos^{-1}\left[\frac{tr(m_R)-1}{2}\right] \quad (3)$$

The unit vector $\hat{a}$, which defines the axis of rotation of the matrix m_R, represents the direction of the retardance vector in the Poincaré sphere. Consequently, this vector is directly related to the orientation of the fast optical axis of the retarder. The fast axis can thus be determined from the direction of $\hat{a}$, as expressed in Eq. (4) [3].

$$a_i = \frac{1}{2\sin R}\sum_{j,k=1}^{3}\epsilon_{i,\,j,\,k}(m_R)_{jk} = \frac{1}{2\sin R}\begin{bmatrix} R_{12} - R_{21} \\ R_{20} - R_{02} \\ R_{01} - R_{10} \end{bmatrix},\ \hat{a} = \frac{a}{\|a\|} = \begin{bmatrix} a_Q \\ a_U \\ a_V \end{bmatrix} \quad (4)$$

To visualize the retardance vector field as a two-dimensional projection, only the a_Q and a_V components of the rotation axis vector $\hat{a}$ were considered. This selection corresponds to projecting the retardance direction onto the Q-V plane of the Poincaré sphere, thereby facilitating the representation of the in-plane orientation of the samples.

Table S1. Properties of CNC suspensions and pitch values of the cholesteric CNC matrix as a function of different tip sonication durations.

Tip sonication duration (minute)	*Properties of CNC suspension*			*Pitch of the dried films (nm)*	*Structural coloration*
	pH	*Conductivity ($\mu S\ cm^{-1}$)*	*Zeta potential (mV)*		
1	*7.01 ± 0.03*	*348 ± 1*	*− 49 ± 2*	*280 ± 41*	*Blue*
5	*6.95 ± 0.02*	*420 ± 2*	*− 51 ± 2*	*388 ± 30*	*Green*
10	*6.89 ± 0.02*	*713 ± 1*	*− 48 ± 1*	*510 ± 35*	*Red*
20	*6.79 ± 0.01*	*1612 ± 1*	*− 52 ± 2*	*820 ± 40*	*Transparent (NIR)*

Comparison of CD between our work and other plasmonic-biopolymer hybrid composite systems

Biological polymers with helical geometry or the ability to form chiral helical templates, including polypeptides, proteins, DNA, and polysaccharides, have been widely used as chiral templates for bottom-up assembly of plasmonic nanoparticles. These plasmonic-biopolymer composite systems can display plasmonic CD due to dipole-dipole interactions between achiral plasmonic NPs spatially distributed in helical configuration. However, Achieving high CD in these conventional is difficult because it requires simultaneous optimization of tightly coupled parameters including chiral geometry, particle size and resonance energy, all while navigating numerous chemical and physical constraints. Our study introduces a different approach using stratified optical media with defined LB- and LD-active optical components. This method decouples these complex parameters and simplifies the materials design process, while producing a higher CD magnitude than the previous plasmonic- biopolymer studies.

In addition, it is important to distinguish our results from studies utilizing the cholesteric CNC to template helical plasmonic nanoparticle assemblies. In many CNC-based systems, CD signals are often reported within the PBG, which may stem from structural pitch modifications rather than intrinsic plasmonic chirality. To ensure a rigorous comparison, we have focused specifically on representative systems where the CD response originates solely from the plasmonic resonance region.

Table S2. Overview of plasmonic CD values of the representative plasmonic-biopolymer systems.

Plasmonic CD			
Peak position (nm)	Absolute values (mdeg)	Hybrid plasmonic-biopolymer systems	Ref.
750	1217	Our work	
579	1.7	Gold nanoparticles attached onto surface of individual CNCs	4
527	260	Gold nanospheres dispersed in cholesteric CNC matrix	5
745	400	DNA nanorods assembly	6
680	200	DNA nanorods assembly	7
740	160	DNA nanorods assembly	8
545	43	Particle double helices (peptide)	9
740	17	Chiral amyloid templates with gold nanorods	10
645	38	Gold nanorods and bovine serum albumin	11

Estimation of equivalent gold atom concentration in AuNP Suspensions.

To estimate the equivalent gold atom concentration from the measured UV-vis spectrum, we first determined the nanoparticle molarity using the Beer-Lambert law according to the reported method [12]. The absorbance at 450 nm was 0.246 with a 1 cm path length, and using the reported extinction coefficient for 30 nm Au nanospheres ($\varepsilon \approx 1.96\times109$ M^{-1} cm^{-1}) [12], the nanoparticle concentration was calculated as 1.26×10^{-7} M (Figure S1). Each AuNP with a diameter in 30 nm contains approximately 8.3×105 mol of Au atoms. Multiplying the nanoparticle molarity by this factor yields an equivalent gold atom concentration of 0.1 mM AuNPs. This value represents the molar concentration of gold atoms present in the colloid, rather than the nanoparticle number concentration. Considering that the measured sample was diluted 5000-fold from the stock solution, the mother suspension corresponds to about 500 mM in gold atom concentration. For template-assisted self-assembly, this concentrated colloid was further diluted with the dispersion solvent to fix the concentration AuNP suspensions at 50 mM for fabrication of plasmonic arrays.

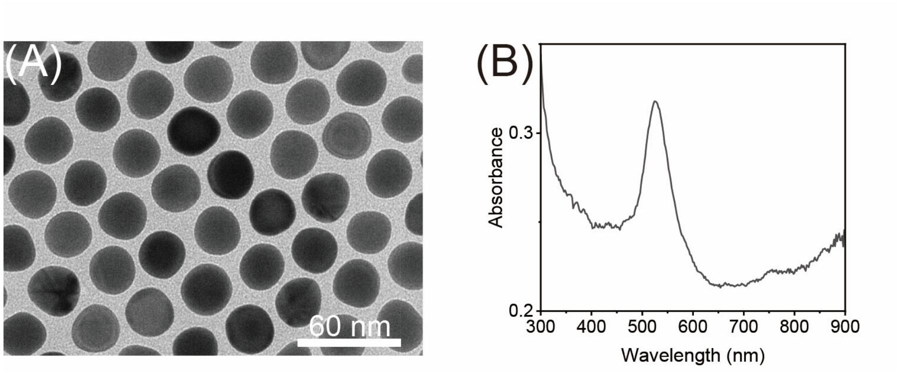

Figure S1. (A) TEM image of AuNPs with an average diameter of 30 nm. (B) UV-vis spectrum of an AuNP suspension showing a LSPR peak at 535 nm, characteristic of 30 nm AuNPs in aqueous medium.

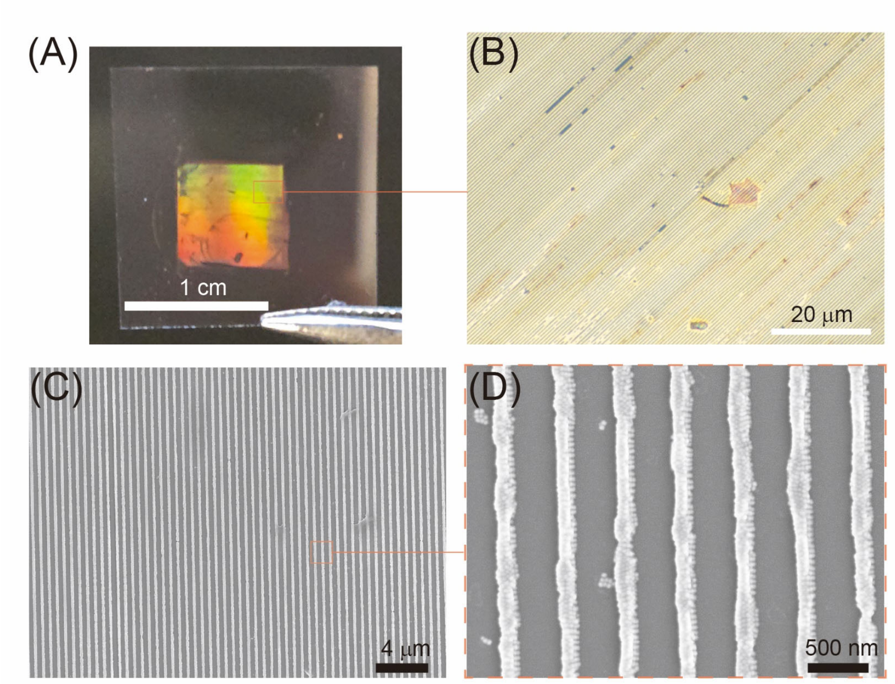

Figure S2. Iridescent appearance and nanostructure of the plasmonic array with a lattice spacing of 500 nm on a glass substrate. (A) A centimeter-scale plasmonic array displays vivid iridescent colors under normal incidence. (B) Optical microscopy image in reflection mode showing large-scale periodical AuNP chains. (C) SEM image resolving the linearly assembled AuNP chains across the substrate. (D) Higher-magnification SEM image revealing a three-layer stacking of nanoparticles within individual chains, with a periodicity of approximately 500 nm.

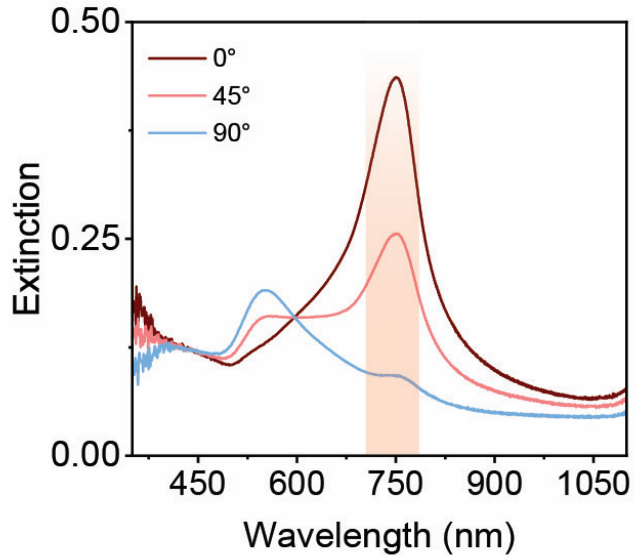

Figure S3. Anisotropic optical properties of the plasmonic array with a lattice spacing of 500 nm. Optical anisotropy at the 750 nm was quantified using the linear dichroic ratio: $LD = 2(A_{\perp} - A_{\parallel})/(A_{\perp} + A_{\parallel})$, where $A_{\parallel}$ and $A_{\perp}$ are extinction values measured at linear polarization angles of 0° and 90°, respectively.

Spectral tuning and mode hybridization of plasmonic resonances in the plasmonic grating

The spectral position (λ) of the SLR depends on the lattice periodicity (Λ) and the refractive index n of the surrounding medium, which can be predicated by $\lambda = n \times \Lambda$ at normal incidence. In our system, due to the asymmetric refractive index at the solid-air interface, scattered light radiates predominantly in the highest refractive index media ($n \approx 1.51$ for CNC/PEG substrate). The asymmetric environment also contributes to the spectral broadening of the resonance features. For lattice spacings of 500 nm and 600 nm, the theoretical SLR positions are at 750 nm and 900 nm, respectively. These values align with the experimental transmittance spectra, where the SLR peaks are spectrally well-separated from the intrinsic LSPR mode.

In contrast, the L400 array exhibits a broad plasmonic dip in its transmittance spectrum. With a lattice spacing of 400 nm, the SLR is predicted to occur at approximately 610 nm. This proximity to the intrinsic LSPR leads to strong spectral overlap and mode coupling (hybridization) between the localized and collective plasmonic modes. Consequently, the resulting feature appears as a single broadened dip that shifts toward the LSPR position when the polarization angle is rotated from 0° to 90° (Figure S4C).

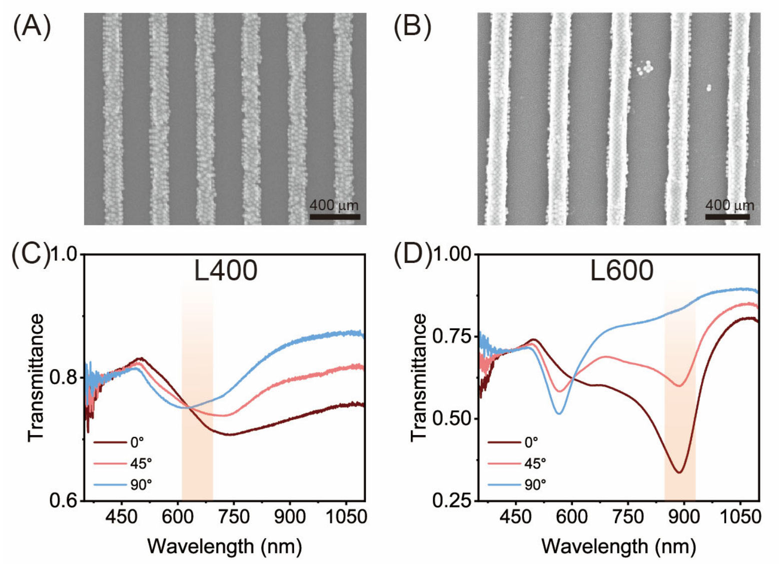

Figure S4. Microstructure and anisotropic optical properties of the plasmonic arrays with varying lattice spacings of 400 and 600 nm. SEM image of the plasmonic array with a lattice spacing of 400 nm (A) and its transmittance spectrum (C). SEM image of the plasmonic array with a lattice spacing of 600 nm (B) and its transmittance spectrum (D), showing the polarization-dependent SLR at 900 nm.

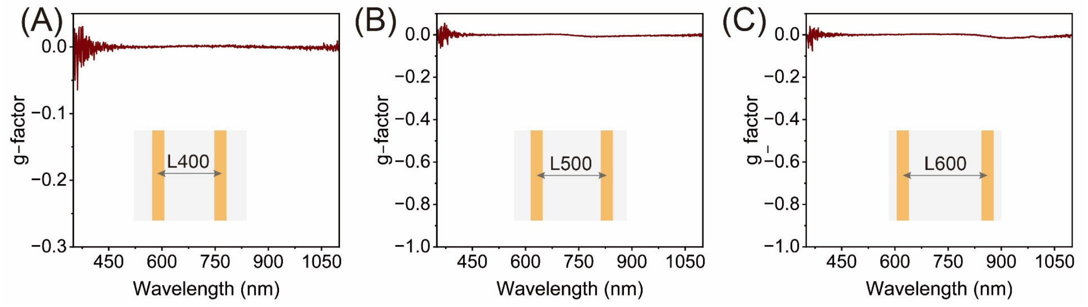

Figure S5. Chiroptical properties of the plasmonic arrays on glass substrates with lattice spacings of 400 nm (A), 500 nm (B) and 600 nm (C). The g-factor values are near-zero for the three samples, indicating the absence of chiroptical activity.

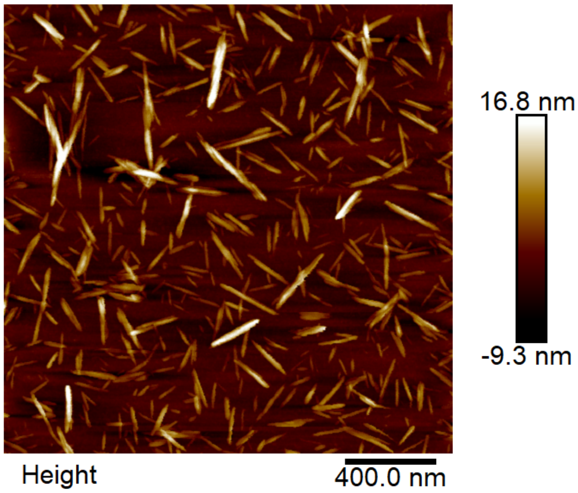

Figure S6. AFM image of the sulfated CNCs, indicating a rodlike morphology.

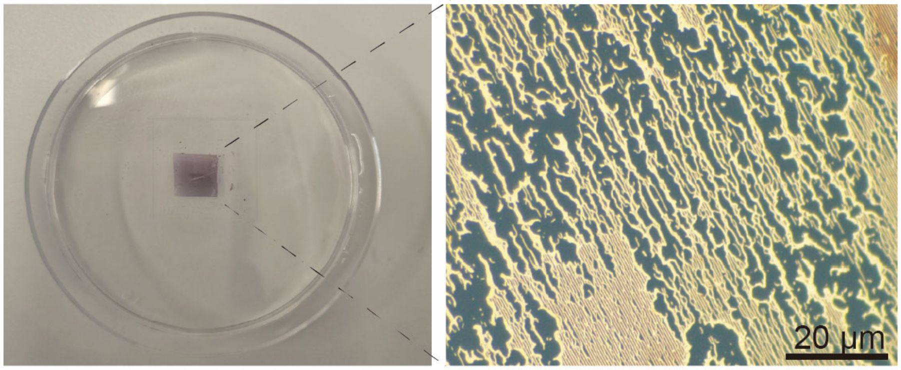

Figure S7. Digital photograph (left side) and optical microscopy image (right side) reveal that the aqueous CNC/PEG suspension can disrupt the AuNP assemblies on a glass substrate in the absence of annealing treatment.

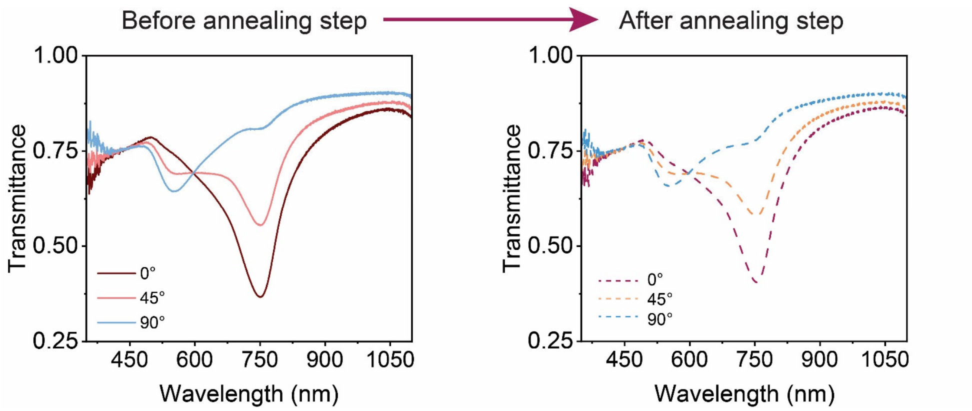

Figure S8. Transmittance spectra of the plasmonic array with a lattice spacing of 500 nm on a glass substrate before and after overnight annealing treatment at 100 °C, exhibiting identical plasmon resonance responses.

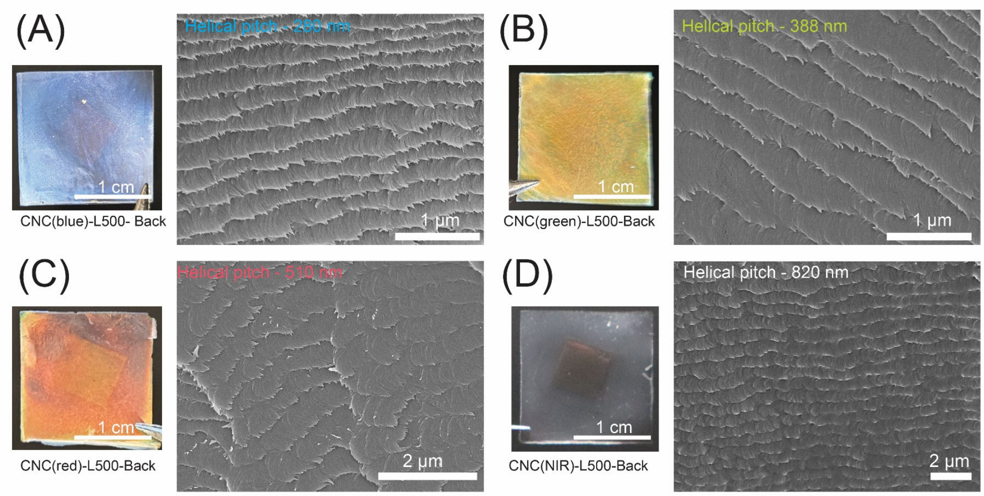

Figure S9. Digital photographs of the plasmonic nanocellulose composites viewed from the non-patterned surface, and SEM cross-section images of the hybrid films. A series of CNC(x)-L500 films exhibit tunable structural colors: blue (A), green (B), red (C) and NIR (D), corresponding to the different pitch values within the cholesteric CNC matrix.

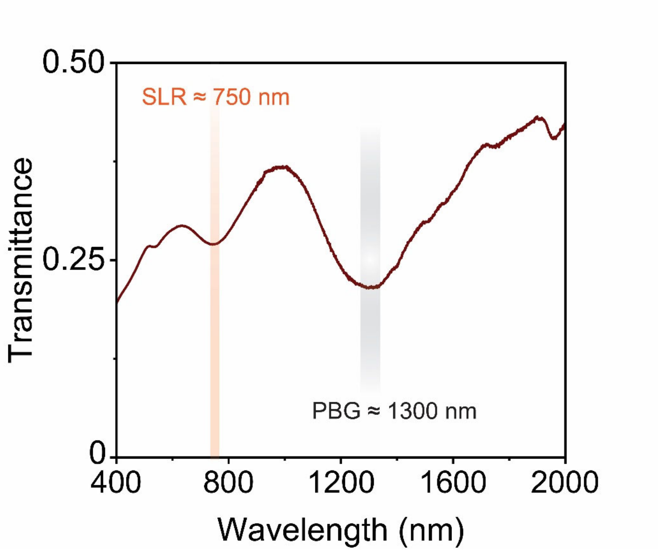

Figure S10. UV-vis-NIR transmittance spectrum of the hybrid film CNC(NIR)-L500 under unpolarized light illumination, indicating emergence of the PBG at 1300 nm (in a gray rectangle), along with the SLR at 750 nm (in an orange rectangle).

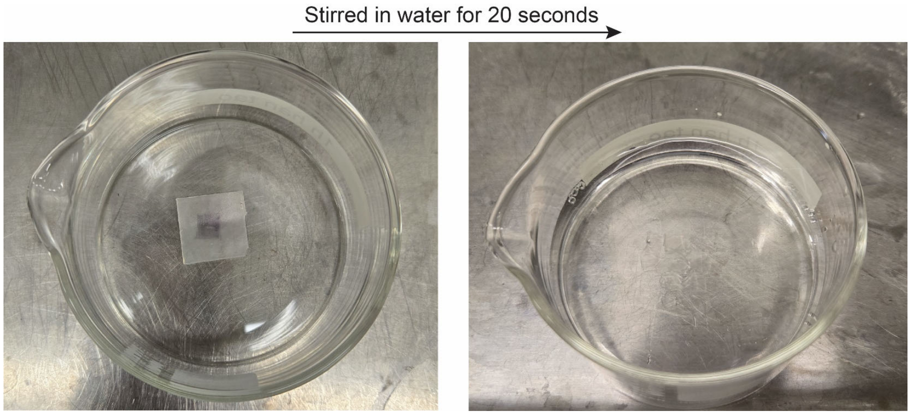

Figure S11. Digital photographs show that the plasmonic nanocellulose film can be dissolved in water after stirring for 20 seconds.

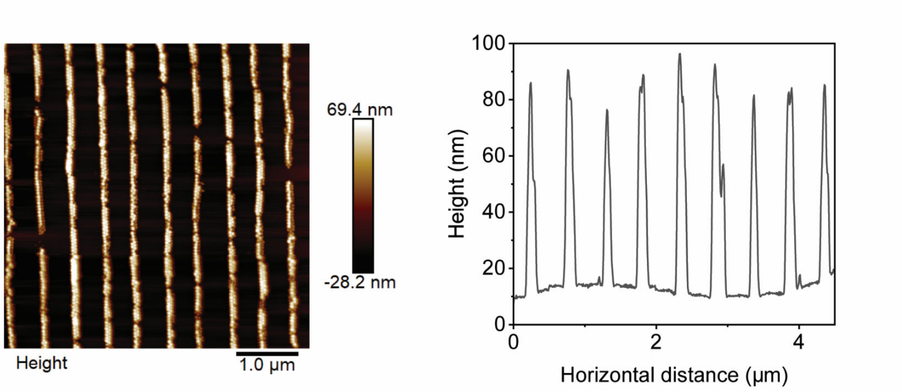

Figure S12. (A) AFM image of the plasmonic array with a lattice spacing of 500 nm on a glass substrate. (B) The measured height profile of AuNP chains is approximately 89 ± 2 nm, suggesting the formation of a three-layer AuNP stacking.

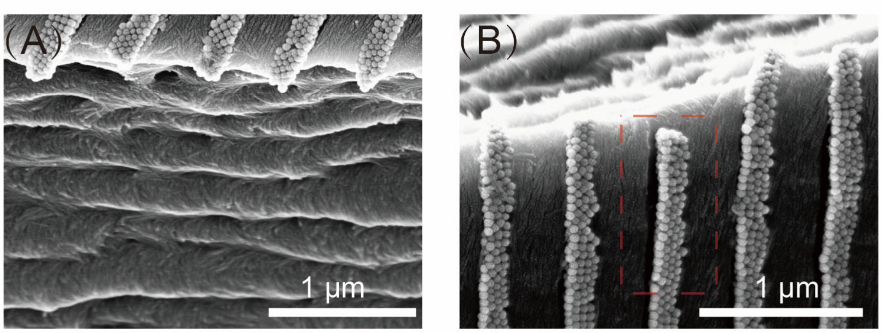

Figure S13. SEM images of the interfacial region of the hybrid film CNC (NIR)-L500: showing no discrete gold nanoparticles within the cholesteric CNC matrix (A) and the AuNP chains remain intact after being transferred to the surface of the composite (B).

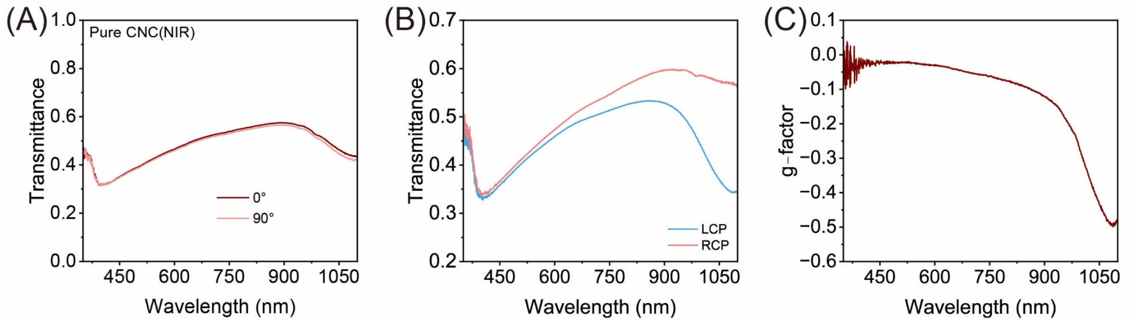

Figure S14. (A) Transmittance spectra of the pure CNC(NIR) film measured under linear polarization at 0° and 90°, indicating negligible linear dichroism. (B) Circularly polarized transmittance spectra of the pure CNC(NIR) film reveal gradually increasing differences between LCP and RCP starting from about 500 nm, which become more pronounced approaching the PBG. (C) The corresponding g-factor profile shows non-zero chiroptical response even in the visible light range, which is attributed to non-uniform helical pitch values within the cholesteric CNC matrix.

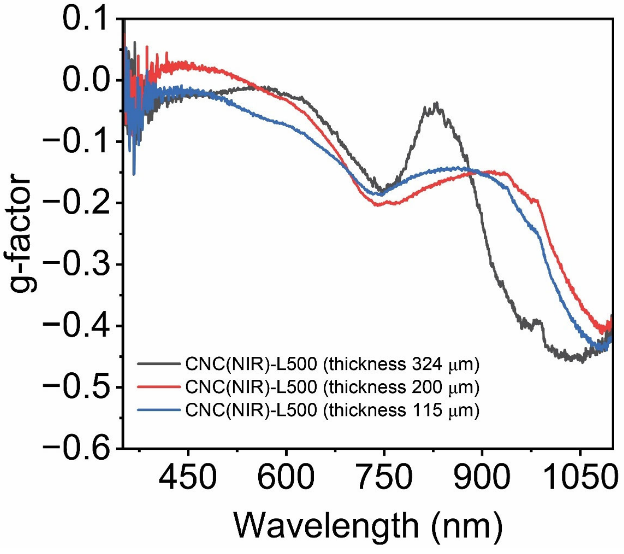

Figure S15. g-factor spectra of the CNC(NIR)-L500 film with varying film thickness.

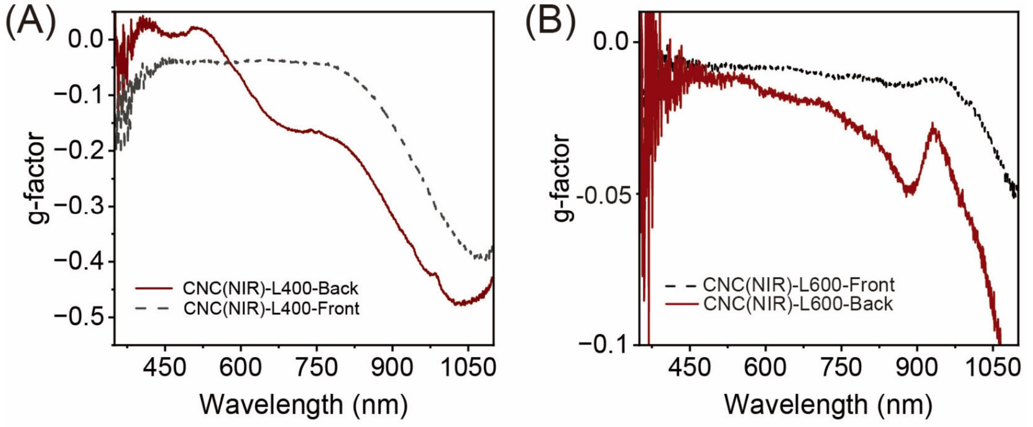

Figure S16. g-factor profiles of hybrid films CNC(NIR)-L400 (A) and CNC(NIR)-L600 (B). Varying the lattice spacing from 400 to 600 nm tunes the spectral position of the plasmonic CD signals.

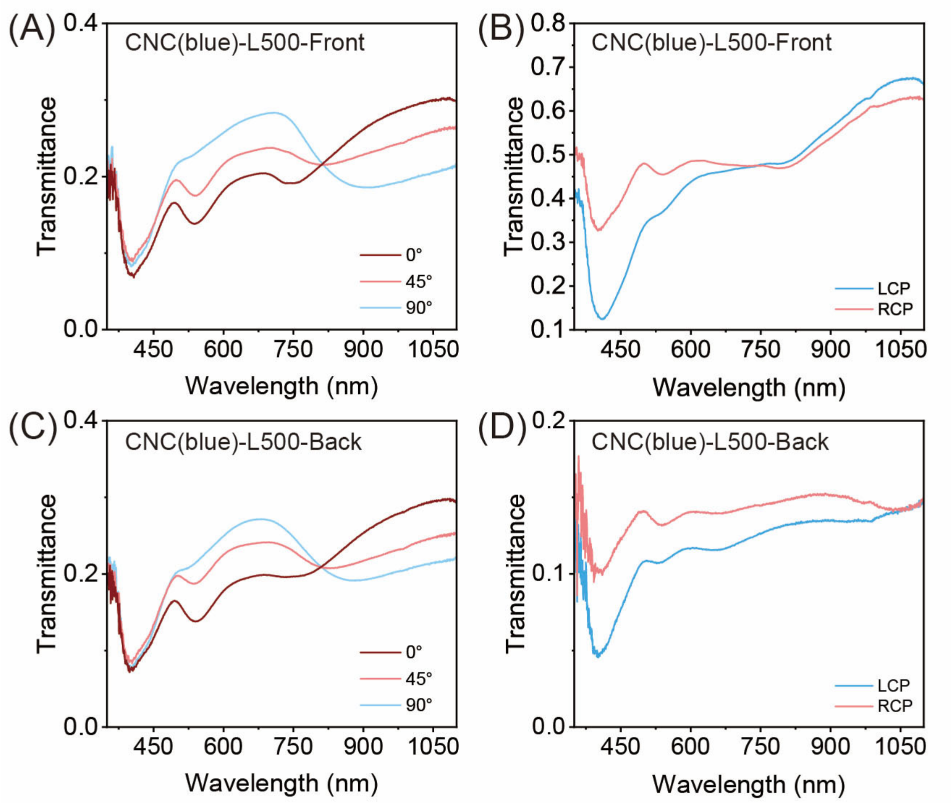

Figure S17. Optical properties of the hybrid film CNC(blue)-L500. Under frontside illumination, transmission spectra of the CNC(blue)-L500 measured at linear polarization angles of 0°, 45°, and 90° (A), and the transmitted LCP/RCP spectra (B). Under backside illumination, transmission spectra of the CNC(blue)-L500 measured under 0°, 45°, and 90° linear polarizations (C), and the transmitted LCP and RCP spectra (D).

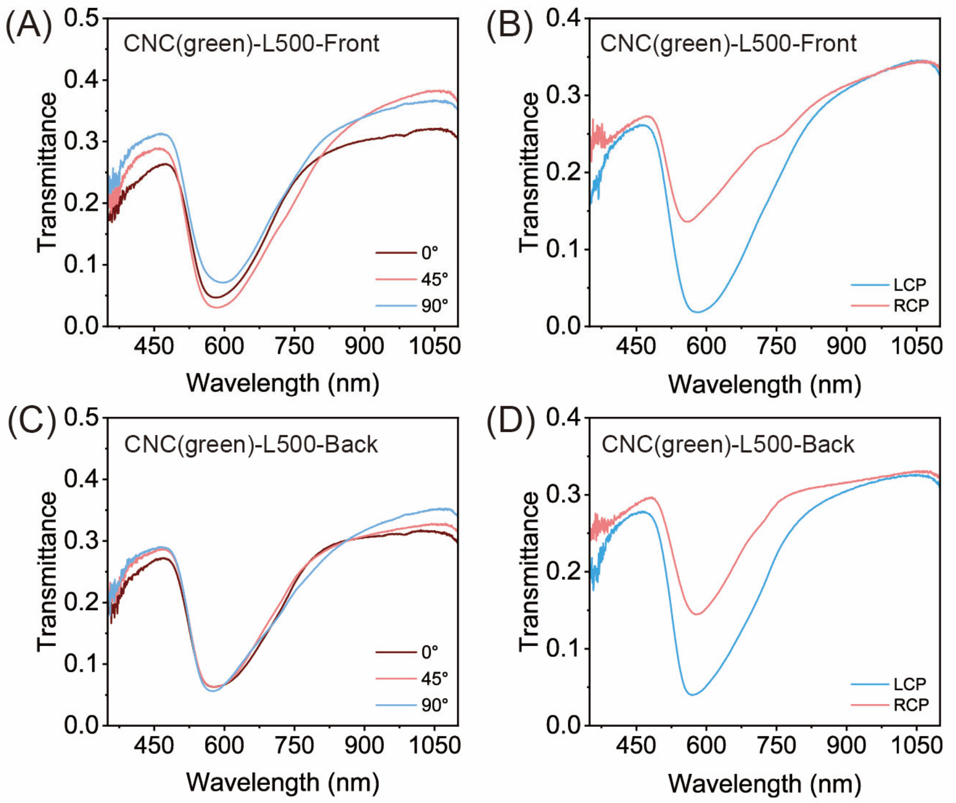

Figure S18. Optical properties of the hybrid film CNC(green)-L500. Under frontside illumination, transmission spectra of the CNC(green)-L500 measured at linear polarization angles of 0°, 45°, and 90° (A), and the transmitted LCP/RCP spectra (B). Under backside illumination, transmission spectra of the CNC(green)-L500 measured under 0°, 45°, and 90° linear polarizations (C), and the transmitted LCP and RCP spectra (D).

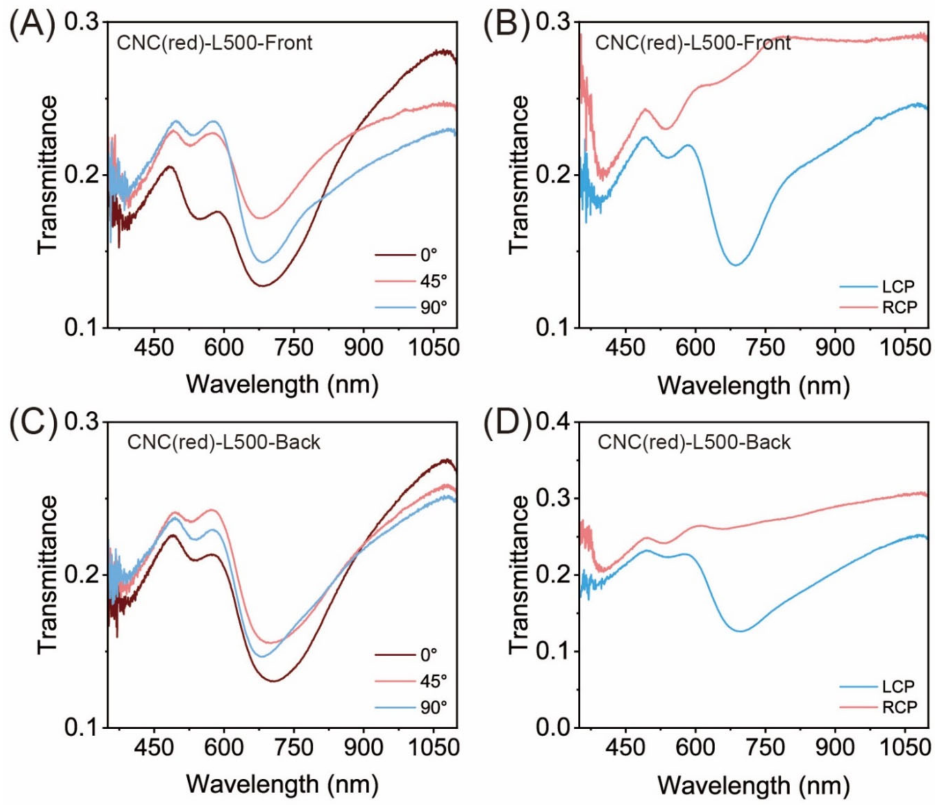

Figure S19. Optical properties of the hybrid film CNC(red)-L500. Under frontside illumination, transmission spectra of the CNC(red)-L500 measured at linear polarization angles of 0°, 45°, and 90° (A), and the transmitted LCP/RCP spectra (B). Under backside illumination, transmission spectra of the CNC(red)-L500 measured under 0°, 45°, and 90° linear polarizations (C), and the transmitted LCP and RCP spectra (D).

Polarimetric Analysis of the hybrid film CNC(blue)-L400 measured at 633 nm:

The MM polarimetry results of the pure CNC(blue) film at 633 nm clearly suggested an absence of dichroic behavior, as evidenced by the first row (diattenuation vector) and the first column (polarizance vector), both of which exhibit null values (Figure S20a). In addition, the sample showed near-zero M_{03}, indicating no CD at 633 nm (Figure S20b). The magnitude of the diattenuation vector, known as the diattenuation D, quantifies the dependence of a sample's transmission on the polarization state of the incident light. Conversely, the magnitude of the polarizance vector, known as the polarizance P, measures the ability of a sample to polarize unpolarized light. To quantitatively assess this hypothesis, the calculated images for the diattenuation D and polarizance P showed values that are practically zero across the entire image (Figure S20c and d). The average values computed for these images are D = 0.0714 and P = 0.0623. Considering that both D and P can range from 0 (non-dichroic material) to 1 (purely dichroic material), these results confirmed the absence of dichroic response in the CNC substrate.

The MM polarimetry results of the hybrid film CNC(blue)-L400 under frontside illumination (Figure S21a), unlike the previous case, exhibited some dichroic behavior, as indicated by non-zero values in the coefficients M_{01} and M_{10} (blue color in contrast to black, which indicates zero value). These elements describe the sample's ability to exhibit linear diattenuation and polarizance, respectively, along the 0° and 90° directions. In other words, due to the presence of absorption anisotropy along these two directions (element M_{01}, associated with the first component of the diattenuation vector), the sample also exhibited the ability to polarize light along the 90° direction (M_{10}, associated with the first component of the polarizance vector). Figure S21b and c show calculated images for the linear diattenuation D and circular retardance. The results suggested that when illuminating the sample from the AuNP array side, the sample presented certain dichroic response. The value computed for linear diattenuation D = 0.30 represents a dichroic enhanced behavior around 30%.

When the same sample was measured under the backside illumination, a response was observed in the CNC(blue)-L400-Back that shares certain similarities with the measurement from the backside (Figure S22a). Similarly, it exhibited linear dichroism (non-zero M_{01} and M_{10} elements as well as a potential retarding-depolarizing behavior, as evidenced by the non-zero values in the 3×3 submatrix. To quantify this analysis, we once again calculated the diattenuation D. The CNC(blue)-L400-Back present certain dichroic response with a linear diattenuation D = 0.24 and (Figure S22b).

On the other hand, both samples exhibit values in the circular retardance (Figure S21C and Figure 22C), which also contributes to the birefringent chirality of the samples, with average values around 10°. However, unlike the previous case (diattenuation), the birefringent response of the two samples is very similar, with global, linear, and circular retardance values being closely matched (54.7° vs 50.58°, 53.86° vs 50.74°, and 10.10° vs 9.85°, respectively). This indicates that the circular retardance component (as well as the other types) is essentially equivalent in both samples. Although it contributes to their chirality, it is the diattenuation effect that plays a significant role in differentiating them.

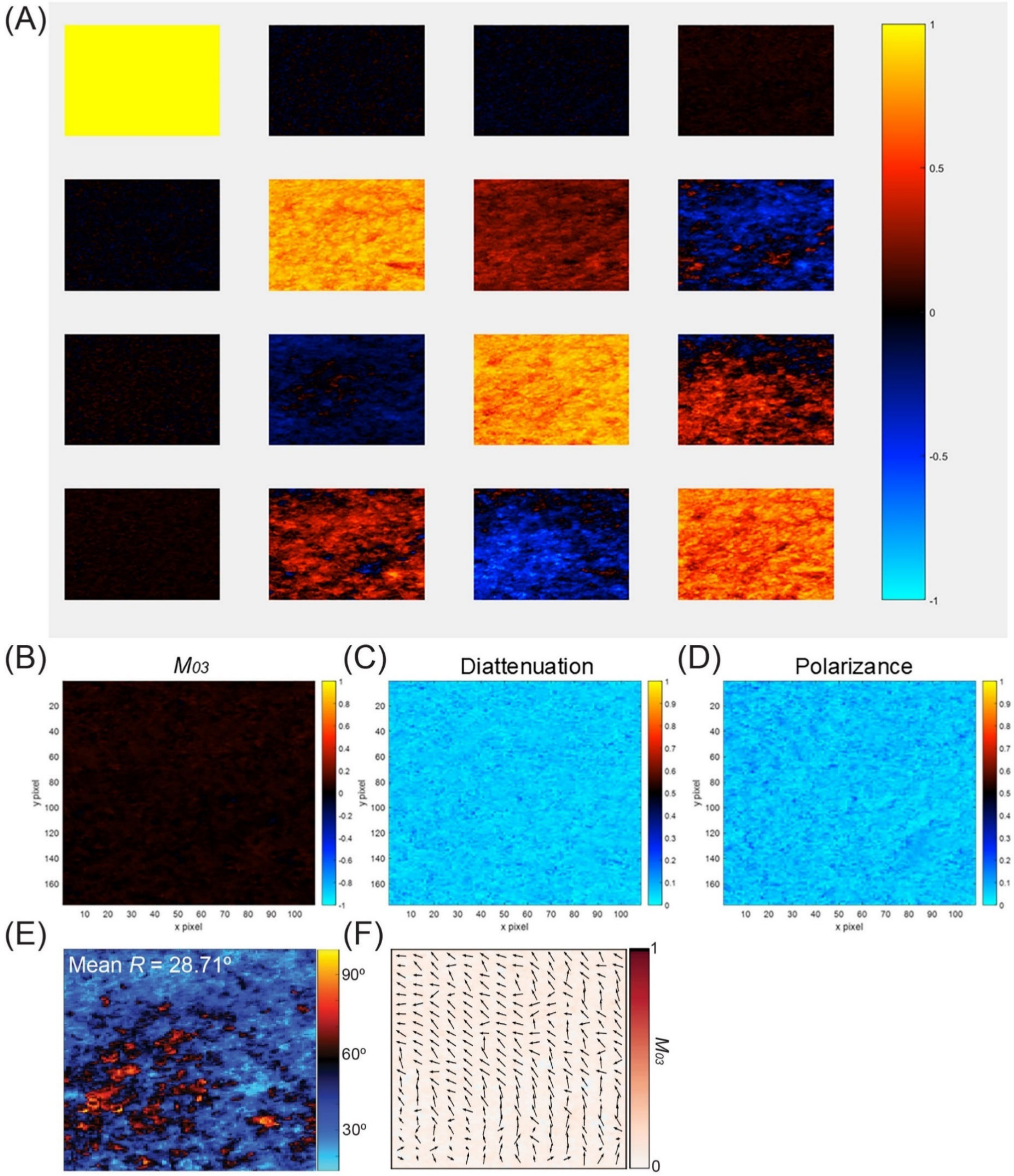

Figure S20. (A) MM polarimetry results for the pure CNC(blue) film at 633 nm. Calculated images for the circular dichroism (B) diattenuation (C) and polarizance (D). The phase retardance map of the pure CNC film (E) and its fast axis field (F).

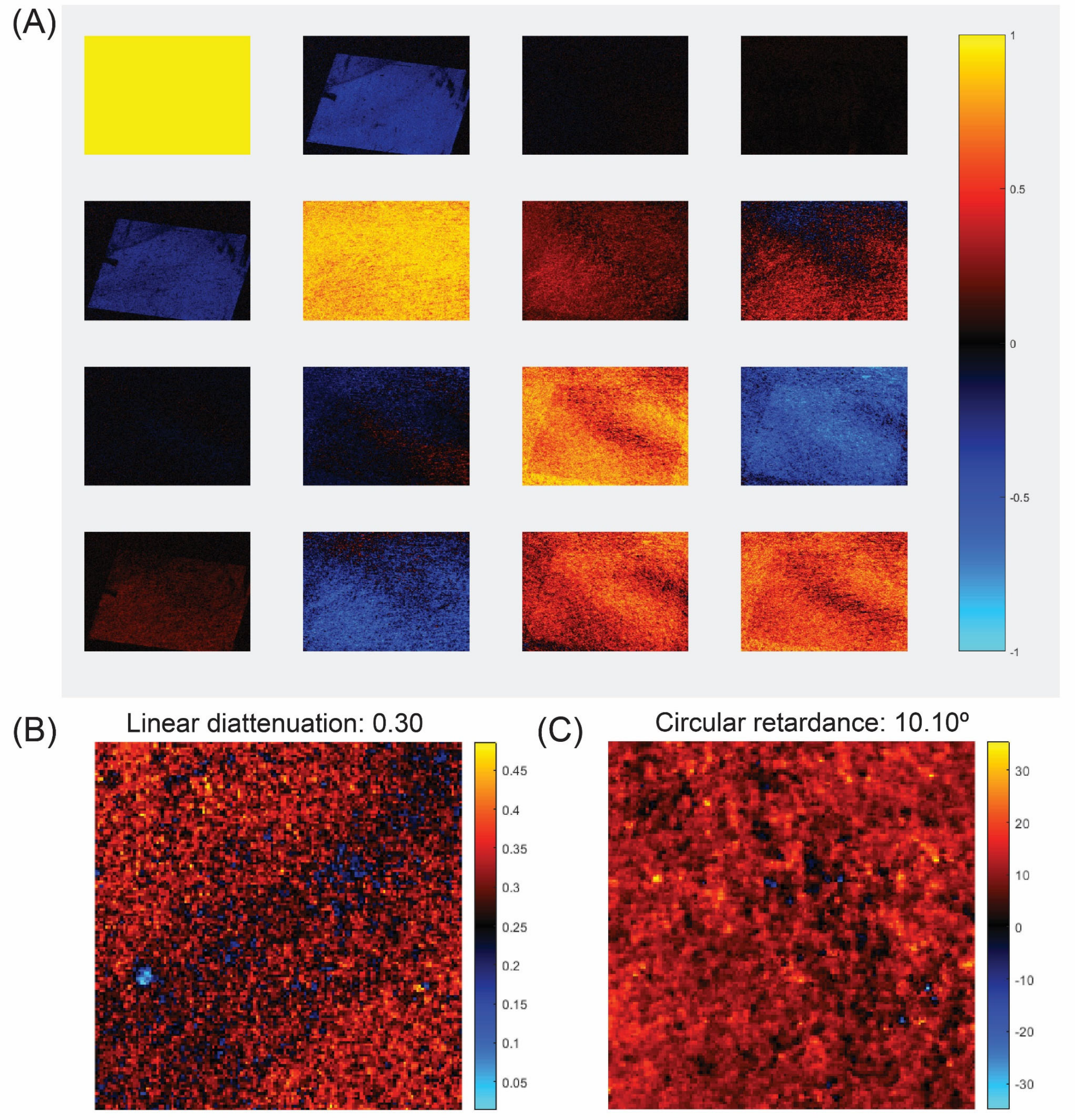

Figure S21. (A) MM polarimetry results for the CNC(blue)-L400 at 633 nm under frontside illumination. Calculated images for the linear diattenuation (B) and circular retardance (C).

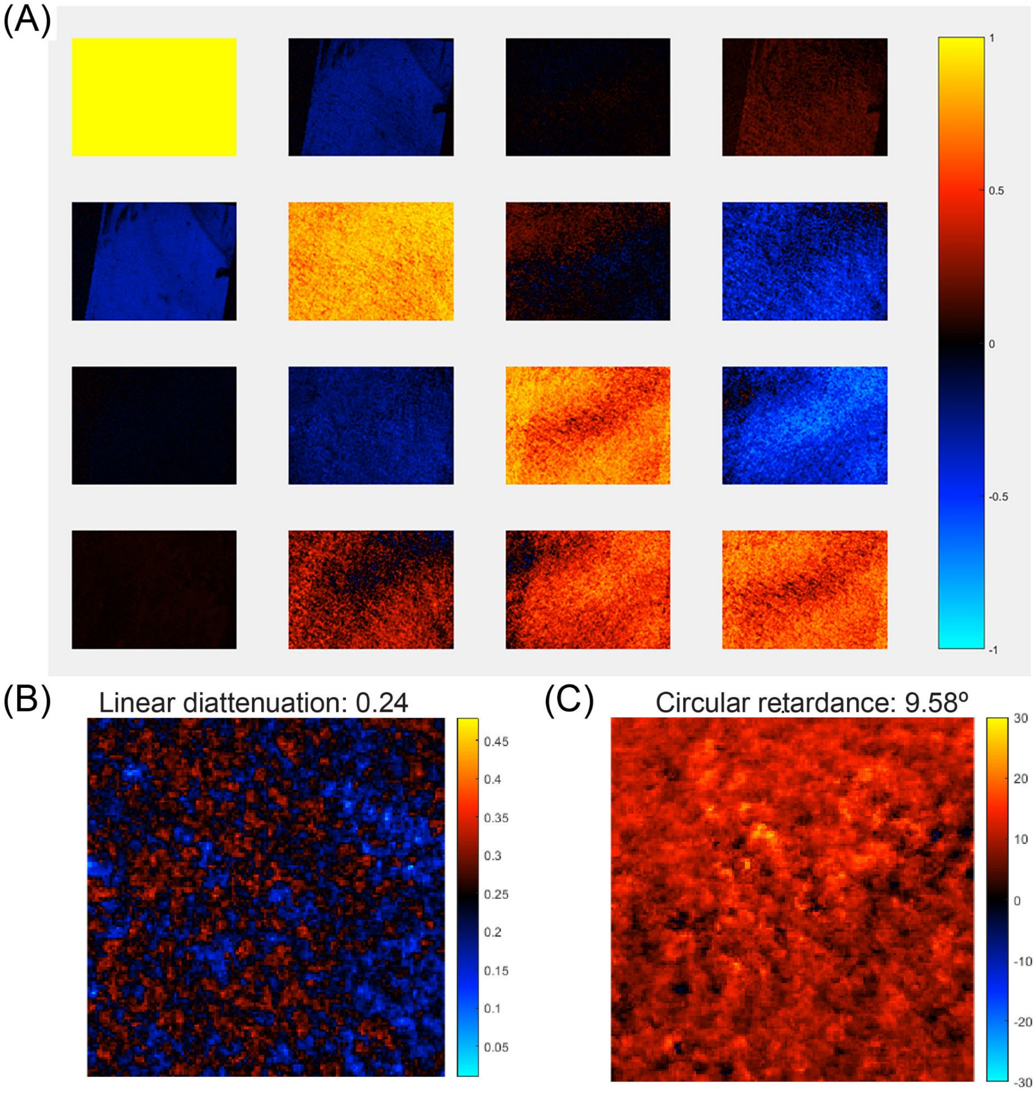

Figure S22. (A) MM polarimetry results for the CNC(blue)-L400 at 633 nm under backside illumination. Calculated images for the linear diattenuation (B) and circular retardance (C).

Mechanism of LB-LD coupling in the plasmonic nanocellulose composite.

Circularly polarized light can be decomposed into two orthogonal linear wave components, E_x (aligned with the slow axis of the CNC layer) and E_y (aligned with the fast axis), with an inherent phase difference $\Delta\phi = (\phi_x - \phi_y)$ of +90° for LCP and −90° for RCP along the propagation axis Z (Figure S23A). Under backside illumination, the cholesteric CNC layer that acts as a phase retarder (mean R = 50.74°) introduces an additional phase delay to the slow-axis component E_x. Consequently, the inherent phase differences are modified differently for LCP and RCP (e.g., LCP shifts towards 0° phase difference, while RCP shifts towards −180° (Figure S23A). Due to these differing resulting phase relationships, LCP and RCP are transformed into two distinct elliptical polarization states with their major axes oriented in different directions relative to the fast axis of cholesteric CNC matrix.

These new, distinct elliptical states then encounter the plasmonic array. Based on the coordinate definitions above, the major axis of the elliptical state derived from LCP is oriented within the first and third quadrants (between the fast y-axis and slow x-axis), whereas the major axis of the RCP-derived ellipse lies within the second and fourth quadrants. The plasmonic array acts as a linear polarizer exhibiting LD by preferentially scattering light polarized along its LD axis while transmitting light orthogonal to it. The interference mechanism is critically dependent on the fact that the LB axis of the CNC retarder and the LD axis of the plasmonic polarizer are not aligned. They are offset by the non-coaxial angle α. Due to this specific misalignment angle α, the RCP-derived elliptical state aligns more closely with the transmission axis of the plasmonic arrays (Figure S23B). Conversely, the LCP-derived elliptical state aligns more closely with the LD axis of the plasmonic array, leading to higher attenuation (Figure S23C). This differential transmission of left- versus right-handed light is the fundamental cause of the observed strong plasmonic CD at surface lattice resonances.

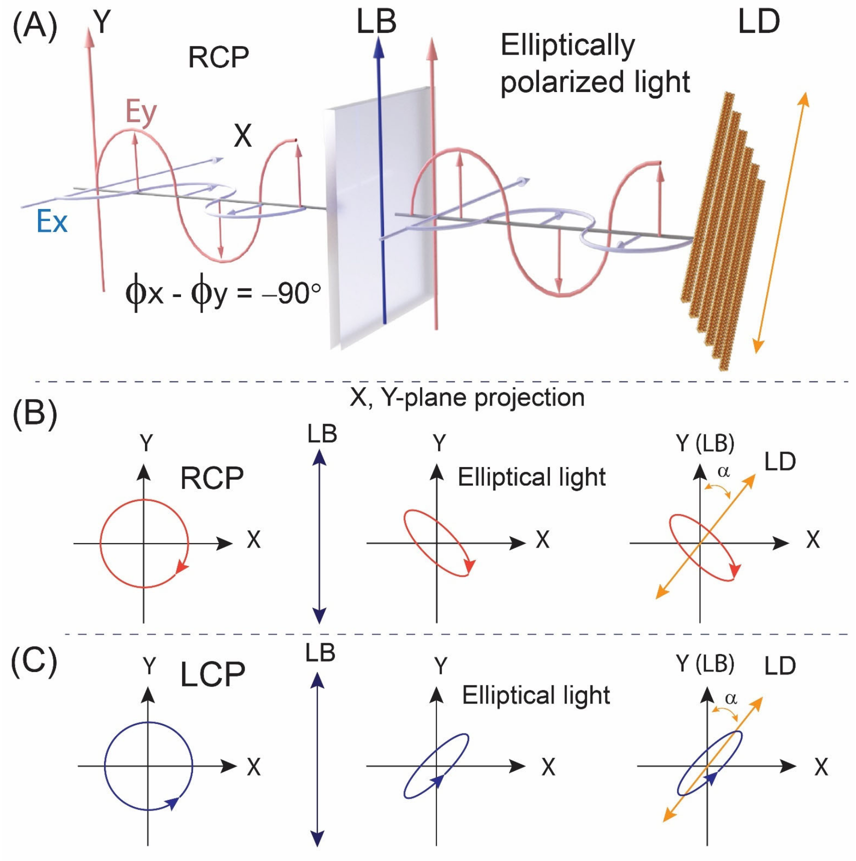

Figure S23. (A) Schematic illustration of RCP that is composed of two or orthogonal linear waves, E_x and E_y, with an inherent phase difference $\Delta\phi = (\phi_x - \phi_y)$ of –90° along the propagation axis Z. The birefringent cholesteric CNC matrix acts as a phase retarder, converting RCP into elliptically polarized that subsequently interacts with the plasmonic array. (B) X, Y-plane projection describes the clockwise rotation of RCP. It shows the orientation of the resulting elliptical state's major axis aligns closely with the transmission axis of the plasmonic array. The offset angle between LB and LD is α. (C) X, Y-plane projection depicting the counterclockwise rotation of LCP. The major axis of the converted elliptically polarized light aligns closely with the LD axis, leading to preferential attenuation of LCP compared to RCP.

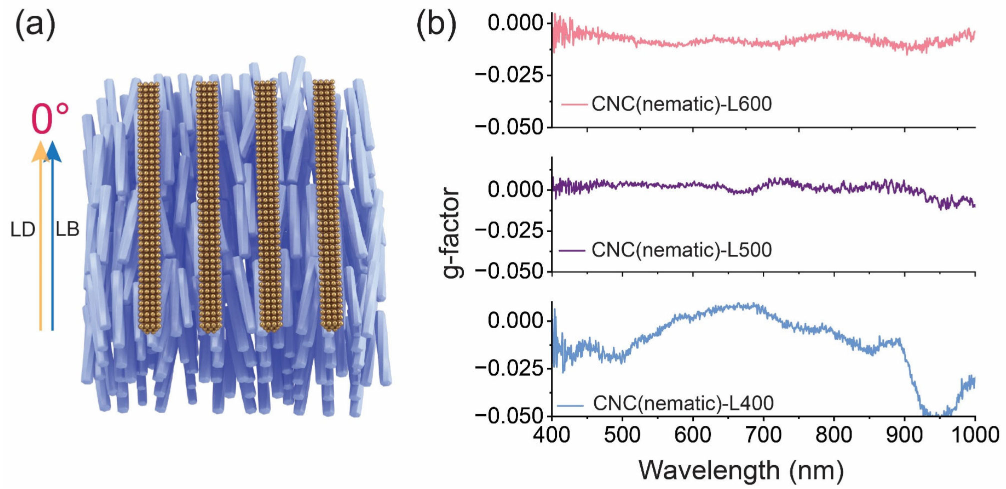

Figure S24. (A) Schematic illustration of the hybrid plasmonic nematic CNC film in which optical axis of the plasmonic array is aligned at 0° relative to the nematic director. (B) The corresponding g-factor profiles of the hybrid CNC(nematic)-L400, L500 and L600 films, exhibiting negligible g-factor values at their respective SLR wavelengths.

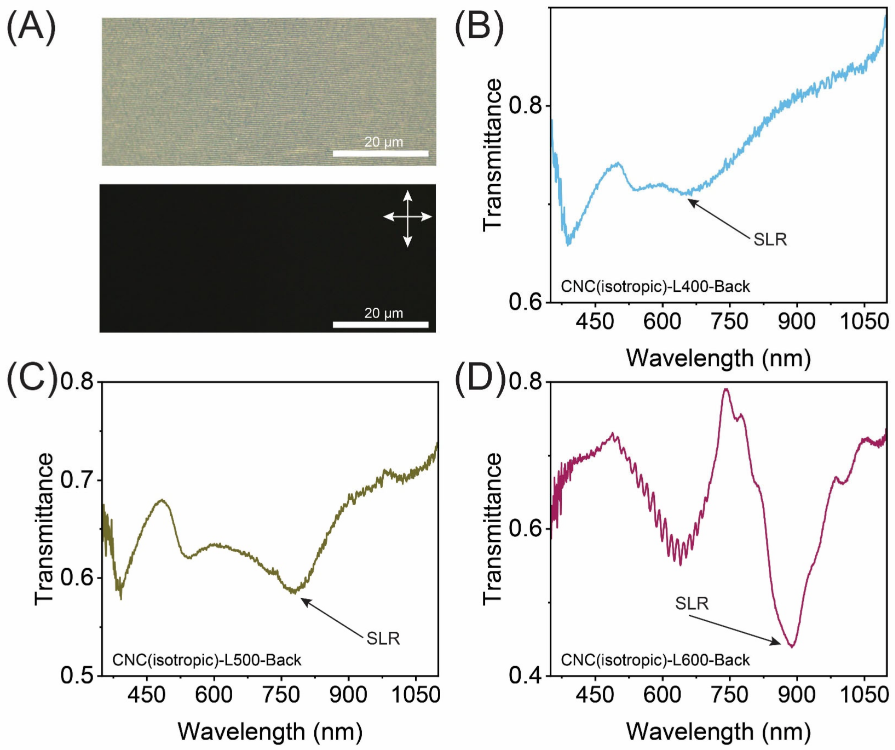

Figure S25. (A) POM images of the hybrid film CNC(isotropic)-L500. The linearly assembled AuNP array is transferred onto the surface of CNC matrix (upper image), while no birefringence is observed under crossed polarizers, confirming the absence of long-range CNC ordering in the matrix. Transmittance spectra of plasmonic CNC(isotropic) composite films with lattice

spacings of 400 nm (B), 500 nm (C) and 600 nm (D), respectively, exhibiting their respective SLR responses.

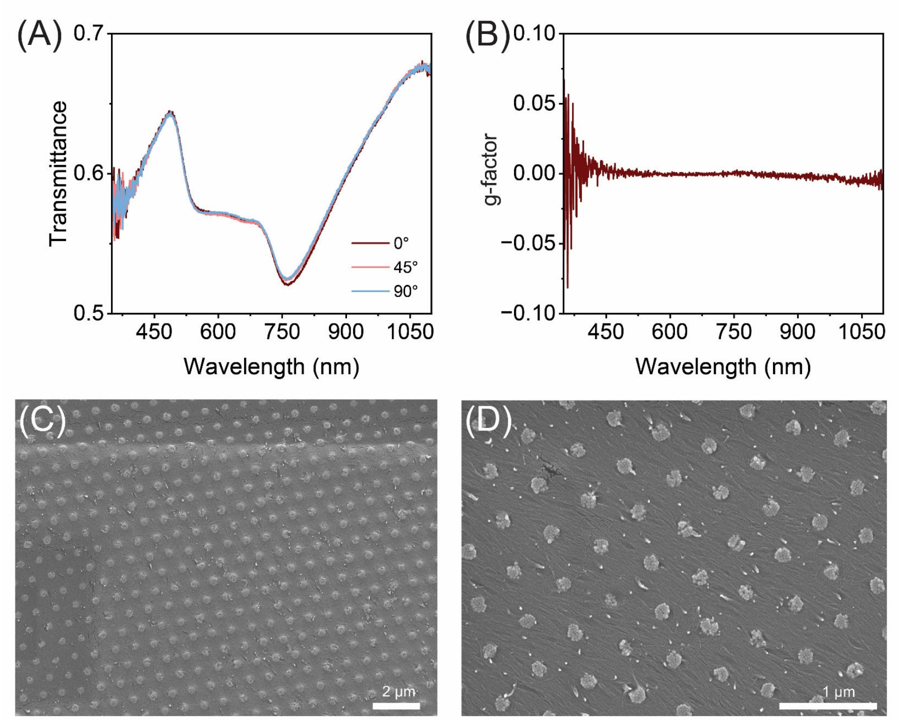

Figure S26. (A) Transmittance spectra of the plasmonic square lattice array (500 nm lattice spacing) on a glass substrate. The SLR peak at 750 nm remains unchanged when the incident linear polarization is rotated from 0°, 45°, to 90°, confirming the absence of linear dichroism. (B) g-factor profile of the plasmonic square lattice array, exhibiting zero g-factor. (C) and (D) SEM images of the CNC(NIR)-H500 sample show a large-scale plasmonic square lattice array on the surface of cholesteric CNC matrix.

References

(1) Tang, Y.; Cohen, A. E. Optical chirality and its interaction with matter. *Phys. Rev. Lett.* **2010**, *104*, 163901.

(2) Zhu, A. Y.; Chen, W. T.; Zaidi, A.; Huang, Y.-W.; Khorasaninejad, M.; Sanjeev, V.; Qiu, C.-W.; Capasso, F. Giant intrinsic chiro-optical activity in planar dielectric nanostructures. *Light Sci. Appl.* **2018**, *7*, 17158–17158.

(3) Lu, S.-Y.; Chipman, R. A. Interpretation of mueller matrices based on polar decomposition. *J. Opt. Soc. Am. A* **1996**, *13*, 1106–1113.

(4) Majoinen, J.; Hassinen, J.; Haataja, J. S.; Rekola, H. T.; Kontturi, E.; Kostiainen, M. A.; Ras, R. H.; Törmä, P.; Ikkala, O. Chiral plasmonics using twisting along cellulose nanocrystals as a template for gold nanoparticles. *Adv. Mater.* **2016**, *28*, 5262–5267.

(5) Chu, G.; Wang, X.; Yin, H.; Shi, Y.; Jiang, H.; Chen, T.; Gao, J.; Qu, D.; Xu, Y.; Ding, D. Free-standing optically switchable chiral plasmonic photonic crystal based on self-assembled cellulose nanorods and gold nanoparticles. *ACS Appl. Mater. Interfaces* **2015**, *7*, 21797–21806.

(6) Kuzyk, A.; Schreiber, R.; Zhang, H.; Govorov, A. O.; Liedl, T.; Liu, N. Reconfigurable 3d plasmonic metamolecules. *Nat. Mater.* **2014**, *13*, 862–866.

(7) Zhou, C.; Duan, X.; Liu, N. A plasmonic nanorod that walks on DNA origami. *Nat. Commun.* **2015**, *6*, 8102.

(8) Wang, M.; Dong, J.; Zhou, C.; Xie, H.; Ni, W.; Wang, S.; Jin, H.; Wang, Q. Reconfigurable plasmonic diastereomers assembled by DNA origami. *ACS Nano* **2019**, *13*, 13702–13708.

(9) Song, C.; Blaber, M. G.; Zhao, G.; Zhang, P.; Fry, H. C.; Schatz, G. C.; Rosi, N. L. Tailorable plasmonic circular dichroism properties of helical nanoparticle superstructures. *Nano Lett.* **2013**, *13*, 3256–3261.

(10) Kumar, J.; Eraña, H.; López-Martínez, E.; Claes, N.; Martín, V. F.; Solís, D. M.; Bals, S.; Cortajarena, A. L.; Castilla, J.; Liz-Marzán, L. M. Detection of amyloid fibrils in parkinson's disease using plasmonic chirality. *Proc. Natl. Acad. Sci. U.S.A.* **2018**, *115*, 3225–3230.

(11) Dominguez-Medina, S.; Kisley, L.; Tauzin, L. J.; Hoggard, A.; Shuang, B.; DS Indrasekara, A. S.; Chen, S.; Wang, L.-Y.; Derry, P. J.; Liopo, A. Adsorption and unfolding of a single protein triggers nanoparticle aggregation. *ACS Nano* **2016**, *10*, 2103–2112.

(12) Ye, X.; Jin, L.; Caglayan, H.; Chen, J.; Xing, G.; Zheng, C.; Doan-Nguyen, V.; Kang, Y.; Engheta, N.; Kagan, C. R. Improved size-tunable synthesis of monodisperse gold nanorods through the use of aromatic additives. *ACS Nano* **2012**, *6*, 2804–2817.
